# Ultrafast Computational Screening of Molecules with Inverted Singlet–Triplet Energy Gaps Using the Pariser–Parr–Pople Semiempirical Quantum Chemistry Method

**DOI:** 10.1021/acs.jpca.3c06357

**Published:** 2024-03-14

**Authors:** Kjell Jorner, Robert Pollice, Cyrille Lavigne, Alán Aspuru-Guzik

**Affiliations:** † Institute of Chemical and Bioengineering, Department of Chemistry and Applied Biosciences, ETH Zürich, Vladimir-Prelog-Weg 1, Zürich CH-8093, Switzerland; ‡ Department of Chemistry and Chemical Engineering, Chalmers University of Technology, Kemigården 4, Gothenburg SE-41258, Sweden; § Chemical Physics Theory Group, Department of Chemistry, University of Toronto, 80 St. George Street, Toronto M5S 3H6, Canada; ∥ Department of Computer Science, University of Toronto, 40 St. George Street, Toronto M5S 2E4, Canada; ⊥ Stratingh Institute for Chemistry, University of Groningen, Nijenborgh 4, Groningen 9747, AG, The Netherlands; # Department of Chemical Engineering & Applied Chemistry, University of Toronto, 200 College Street, Toronto M5S 3E5, Canada; ¶ Department of Materials Science & Engineering, University of Toronto, 184 College Street, Toronto M5S 3E4, Canada; ∇ Vector Institute for Artificial Intelligence, 661 University Ave. Suite 710, Toronto M5G 1M1, Canada; ○ Lebovic Fellow, Canadian Institute for Advanced Research (CIFAR), 661 University Avenue, Toronto M5G 1M1, Canada; ⧫ Acceleration Consortium, University of Toronto, 700 University Avenue, Toronto M5G 1Z5, Canada

## Abstract

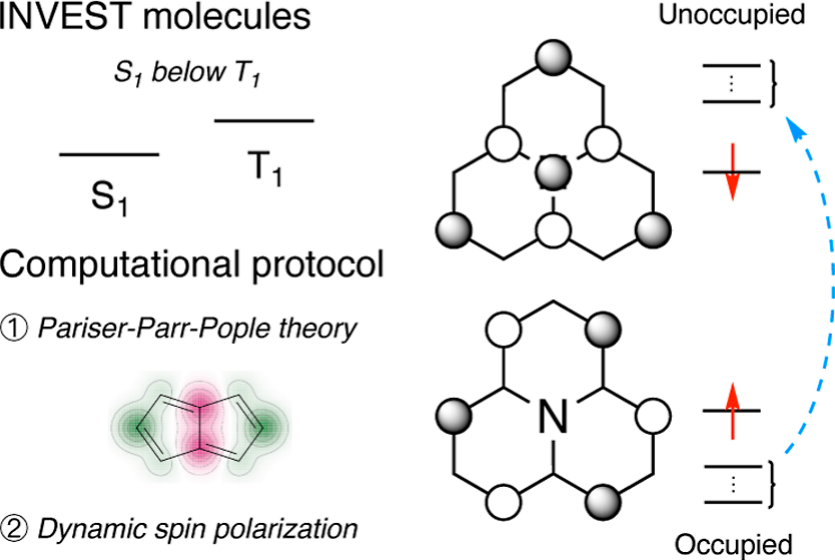

Molecules with an inverted energy gap between their first singlet and triplet excited states have promising applications in the next generation of organic light-emitting diode (OLED) materials. Unfortunately, such molecules are rare, and only a handful of examples are currently known. High-throughput virtual screening could assist in finding novel classes of these molecules, but current efforts are hampered by the high computational cost of the required quantum chemical methods. We present a method based on the semiempirical Pariser–Parr–Pople theory augmented by perturbation theory and show that it reproduces inverted gaps at a fraction of the cost of currently employed excited-state calculations. Our study paves the way for ultrahigh-throughput virtual screening and inverse design to accelerate the discovery and development of this new generation of OLED materials.

## Introduction

Organic light-emitting diode (OLED) is a technology for generating light from electricity using organic molecules.^1^ The first generation of OLEDs was based on fluorescent organic molecules with a maximum efficiency of 25% due to spin statistics. As the transition T_1_ → S_0_, from the lowest excited triplet state to the singlet ground state, is spin-forbidden, the OLED molecule can emit efficiently only from its first excited state of singlet multiplicity, S_1_. The second generation of OLEDs exploited ways of increasing the rate of this spin-forbidden phosphorescence. The third generation of the OLEDs is based on thermally activated delayed fluorescence, where the S_1_ state is partially populated from a near-lying T_1_ state. For this to happen, the gap Δ*E*_ST_ between the states needs to be sufficiently small so that the thermal activation competes with nonradiative decay processes from T_1_. However, as the T_1_ population is still substantial, problems with stability and lower-than-ideal quantum yields due to nonradiative decay persist. The logical step for the next generation of OLEDs is to emit directly from an S_1_ state that lies below the T_1_ state, potentially converting all of the electrically generated excitons into photons, i.e., 100% internal quantum efficiency, while avoiding decay reactions from the T_1_ state.

Molecules with an inverted singlet–triplet energy gap (INVEST) are exceedingly rare.^2^ They violate Hund’s rule of maximum multiplicity as applied to the S_1_ and T_1_ states of molecules, which describes that the electronic state with the highest spin (in this case, the triplet) should be the lowest in energy. While a handful of examples of molecules violating Hund’s rule in the excited state have been known since the 1970s and 1980s,^3−7^ it is only recently that they have received considerable attention for application in OLEDs.^8−10^ As they are rare, recent efforts have used high-throughput virtual screening (HTVS) with computational chemistry to identify new compounds with inverted gaps, focusing on expanding the local chemical space around specific scaffolds^9−15^ or scanning larger (combinatorically or experimentally derived) data sets to identify novel scaffolds.^16−18^ These HTVS efforts have been successful in identifying several new INVEST candidates, some of which have also been synthesized and tested experimentally.^10^

While this early success of HTVS is highly encouraging, it is hampered by the cost of computational methods. Excitation energies are routinely calculated by time-dependent density functional theory (TD-DFT), which has become a workhorse of computational photophysics over the last decades.^19^ Unfortunately, it has been shown that TD-DFT fails to capture the inverted gap of INVEST compounds, as the method only considers single electronic excitations.^8^ Inclusion of at least double excitations is necessary to reproduce the inverted gaps, corresponding to methods such as double hybrid TD-DFT (via perturbation theory)^20^ or excited-state coupled-cluster methods such as second-order approximate coupled-cluster singles and doubles (CC2) or equation of motion coupled-cluster with single and double excitations (EOM-CCSD). In line with the results of benchmarking studies,^12,21^ recent HTVS studies have used methods such as the complete active space self-consistent field (CASSCF) and CC2 for preliminary screening, while confirming inverted gaps with more expensive methods such as the multistate complete active space second-order perturbation theory (MS-CASPT2) and EOM-CCSD. These methods are not only expensive (compared to TD-DFT) but also require the choice of an orbital active space (in the case of the CAS methods), something which is not routinely automatized (although advances have been made).^22,23^

Motivated by the need for faster methods for HTVS of INVEST compounds, we wondered if it would be possible to perform much simpler calculations as a prescreening step for the more expensive methods. Based on the prior work in the literature from the 1970s and 1980s, we find that the inverted gaps can be recovered already with the semiempirical Pariser–Parr–Pople (PPP) method using configuration interaction singles (CIS) and adding key double excitations. The PPP method considers only the π electrons in a minimal valence basis and approximates all integrals from experimental data and a few empirical parameters, making it computationally extremely cheap. At the same time, it retains the conceptual clarity of Hückel theory and allows for a straightforward interpretation of the inverted gap in terms of the well-known concept of dynamic spin polarization (DSP). We show that the method is capable of finding promising hits both in the local chemical space around known scaffolds and of identifying hits in more diverse data sets. While there are clear limitations to the method, we believe that it will open the doors for ultrahigh-throughput virtual screening of compound libraries several orders of magnitude larger than today. The method is also perfectly applicable as a scoring function in inverse design algorithms.

## Theory

While the recent literature has focused on applying high-level ab initio and DFT methods to study INVEST compounds, we believe that some conceptual clarity has been lost in the process. Instead, we apply the simplest possible electronic structure method that can capture the physics of the problem. The PPP method is an extension of the semiempirical Hückel molecular orbital theory for π-electron systems that includes electron correlation.^24−26^ It originated in the 1950s, with new parametrizations being developed mainly in the 1960s and 1970s, and was used in the dye industry at least until the 1980s before the advent of more accurate ab initio methods.^27^ The PPP method retains the conceptual clarity of Hückel theory while at the same time including the electron correlation that is necessary to capture the inverted gap. For another recent application of PPP to INVEST compounds, see the work by Painelli and co-workers.^28^

### PPP Theory

Within Pople’s formulation of the PPP theory, Roothaan’s equations are solved self-consistently.^26^ Under the zero differential overlap (ZDO) approximation, overlap integrals are neglected for orbitals on different centers, leading to a simplification of the Roothaan equations as **S** becomes the unit matrix **I**
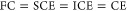
1where **F** is the Fock matrix, **C** contains the molecular orbital coefficients, and **E** is the diagonal matrix of the orbital energy eigenvalues. Given **F**, the corresponding **C** and **E** can then be determined either self-consistently (following Pople^26^) or via configuration interaction (following Pariser and Parr^24,25^), most often starting from a guess **C** from the corresponding Hückel model. The main computational effort is then used to construct **F**, which has the following matrix elements^29^
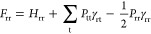
2
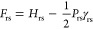
3where *P*_rs_ is the element of the density matrix **P**, and γ_rr_ and γ_rs_ are parameters called the one-center and two-center electron repulsion integrals for centers r and s, respectively. The core resonance integral matrix elements of **H** are given by
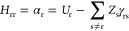
4
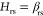
5where α_r_ is the one-center core resonance integral, *U*_r_ is a parameter of the model called the atomic valence-state potential, *Z*_s_ is the effective nuclear charge, and β_rs_ is the two-center resonance integral, another parameter of the model.

### Parameterization

The four parameters are thus *U*_r_, γ_rr_, γ_rs_, and β_rs_. They are derived from experiment, first-principles, or a combination of both. The Pariser–Parr approximation leads to
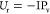
6where IP_v_ is the valence-state ionization potential of the orbital and atom in question (e.g., a 2p orbital of an sp^2^-hybridized C atom).^30^ The IP_*v*_ values (and the corresponding electron affinity EA_v_) are tabulated by Hinze and Jaffé as determined from experimental data.^31,32^ γ_rr_ also enjoys an almost universally adopted approximation, due to Pariser and Parr
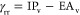
7

For γ_rs_ and β_rs_, there is much less consensus. Formulas for γ_rs_ have been suggested by, among others, Mataga and Nishimoto^33^ and Ohno,^34^ but here, we follow the approach by Beveridge and Hinze^29^
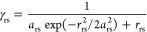
8where *r*_rs_ is the distance between the two atom centers r and s, and
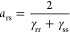
9

For β_rs_, expressions have been given by, e.g., Linderberg^35^ and Jug,^36^ based on first principles and by Dewar^37^ based on experimental data. Here, we again follow Beveridge and Hinze,^29^ who derived the expression as, following Ohno^34^
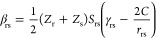
10where *C* = 0.545 is a parameter that was fit in the original publication to reproduce experimental excitation energies.^29^ The overlap integral *S*_rs_ is determined from the overlap of Slater p orbitals with exponents
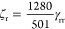
11according to recursion formulas given by Mulliken.^38^ Thus, the four parameters *U*_r_, γ_rr_, γ_rs_, and β_rs_ are expressed completely in terms of the corresponding valence-state ionization potential (IP_v_) and electron affinity (EA_v_) as well as the completely empirical parameter *C*. We have used the set of valence-state ionization potentials and electron affinities from Beveridge and Hinze (Table S1).^29^

The distance dependence of β_rs_ and γ_rs_ allows for the treatment of bond-length alternation beyond idealized geometries. In addition, we have followed the literature^39^ and added an angle dependence to β_rs_ according to
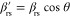
12where θ is the twist angle between the two p orbitals. We have determined θ as the average of all possible dihedrals involving the two atoms *r* and *s*.

### Excited-State Calculations

The energy difference of the vertically excited S_1_ and T_1_ states can be determined by Roothaan’s expression at the SCF level^40^
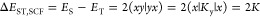
13where *K* is the exchange integral between the two orbitals involved in the single excitation [normally the highest occupied molecular orbital (HOMO) and the lowest unoccupied molecular orbital (LUMO)]. As defined here, a positive Δ*E*_ST_ corresponds to the usual situation with the triplet being lower in energy than the singlet, while a negative Δ*E*_ST_ corresponds to an inverted gap. The exchange integral depends strongly on the spatial overlap between the two orbitals, which in the ZDO approximation can be computed as^41^
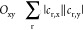
14where |*c*_r,i_| is the absolute value of the coefficient of orbital i on center r. When the overlap is zero, the exchange interaction vanishes, and Δ*E*_ST,SCF_ is zero, and, unfortunately, so is the oscillator strength, *f*, between the S_1_ excited state and the S_0_ ground state,^42^ which is nonoptimal for OLED materials that should emit light.^43^ Consequently, there is a trade-off between having a small HOMO–LUMO overlap to reduce the exchange interaction while still maintaining a sufficient oscillator strength.

**Figure 1:**
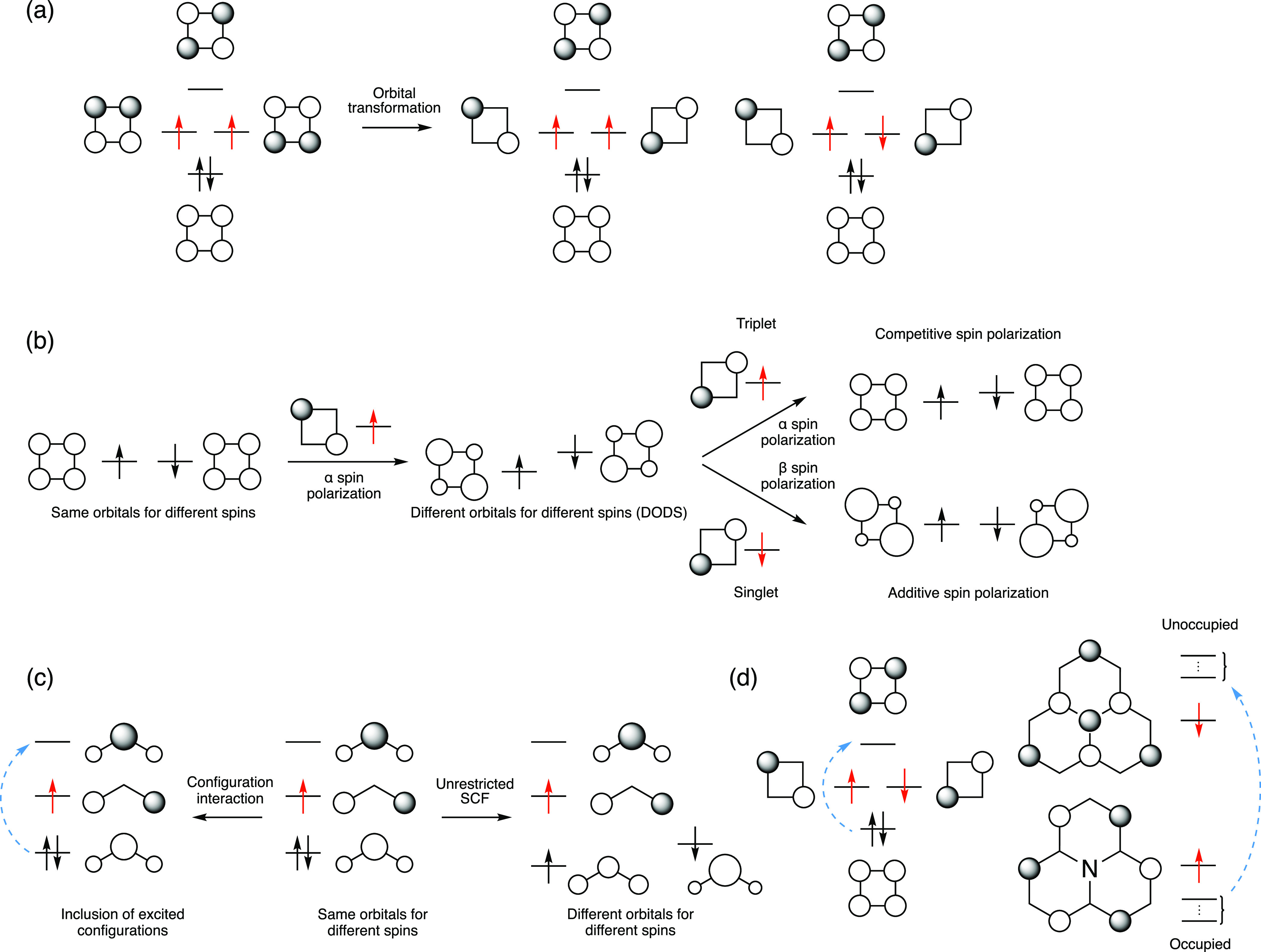
Dynamic spin polarization stabilizes the open-shell singlet state over the open-shell triplet state. (a) Transformation of the canonical frontier molecular orbitals creates a set of disjoint nonbonding molecular orbitals for cyclobutadiene. Singlet and triplet occupations are shown. (b) Dynamic spin polarization preferentially stabilizes the singlet state of cyclobutadiene through additive spin polarization for the singlet and competitive spin polarization for the triplet. Panel (b) has been adapted with permission from ref (46). Copyright 1989 American Chemical Society. (c) An alternative description of the spin polarization phenomenon is described via configuration interaction and admixture of excited configurations into the ground state. (d) In the same way, as cyclobutadiene is stabilized by DSP in the singlet ground state, molecules with inverted singlet–triplet gaps in the excited state are also stabilized by DSP that can be described by the admixture of excited configurations. For singly excited states, this means the addition of doubly excited configurations.

A more precise expression for Δ*E*_ST_ can be obtained by CIS.^29^ While CIS adds some correlation for the excited states, it is necessary to add additional excitations beyond singles to capture the inverted Δ*E*_ST_. Fortunately, the most important excitations have already been identified in the literature long ago. Singlet–triplet inversion occurs also for the ground state, where several violations of Hund’s rule are well known, for example, for bond-equalized cyclobutadiene at the *D*_4h_ geometry. Borden and Davidson explained this effect in terms of DSP,^44^ in which the electrons of a pair of disjoint nonbonding molecular orbitals (Figure 1a) experience stronger electron correlation effects in the singlet state as compared to the triplet state (Figure 1b).^45^ For a pedagogic introduction to the topic, the reader is referred to an excellent article by Karafiloglou.^46^ As outlined by Kollmar and Staemmler,^3^ as well as Malrieu and co-workers,^47,48^ an alternative view of spin polarization is described through the admixture of excited configurations into the electronic wave function of the ground state (Figure 1c).^49−51^ This is shown in Figure 1c for the case of static spin polarization in the allyl radical. The DSP effect for the S_1_ excited state can correspondingly be described by the admixture of doubly excited configurations,^3^ singly excited with respect to the S_1_ state, and doubly excited with respect to the S_0_ ground state (Figure 1d).

As first shown by Kollmar and Staemmler,^3^ and recently re-emphasized by Pernal and co-workers,^52^ the Δ*E*_ST_ can be approximated by a combination of two terms, the first one being the exchange interaction 2*K* (given by eq 13) and a correction term Δ*E*^DSP^ due to the DSP, which can be approximated with the perturbation theory^3^
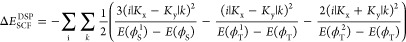
15here, *K*_x_ and *K*_y_ are exchange operators, *E*(ϕ_S_) and *E*(ϕ_T_) are the energies of the singlet and triplet excited determinants, while *E*(ϕ_S_^1^), *E*(ϕ_T_^1^), and *E*(ϕ_T_^2^) are the energies of doubly excited determinants. They can be written in terms of the corresponding orbital energies, ε_i_, provided the same orbitals are used for both singlet and triplet states^47^
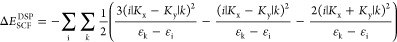
16

The simplest approximation for Δ*E*_ST_ taking DSP into account is then
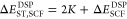
17

Alternatively, Δ*E*^DSP^ can be calculated with respect to the CIS states^53^
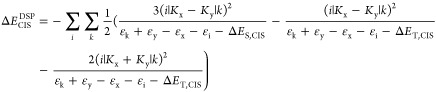
18and added as a correction to the energy gap at the CIS level to yield a possibly more accurate value
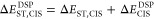
19

## Methods

The PPP method was implemented in the Python package Coulson,^54^ which is freely available on GitHub with a permissive open-source MIT license. Further details on Coulson will be reported elsewhere. The PPP wave function was converged with the self-consistent field method using the naive variational principle. CIS calculations used the converged SCF wave function as the reference and modeled vertical excitations based on the precomputed geometries. To make the calculations more robust, we derived a spline interpolation of the overlap integrals,^38^ which will be reported separately. Evaluated on the azaphenalene data set (vide infra), it shows good accuracy with *R*^2^ = 1.000 and RMSE = 0.004 eV for Δ*E*_ST,CIS_^DSP^ (Figure S1). All calculations were performed on a MacBook Air laptop computer with an M2 processor. We further integrated Coulson with PySCF(55) to provide alternative algorithms for the SCF convergence and CIS and found the results consistent within numerical accuracy. To give an indication of the computational cost, the SCF calculation for azaphenalene (13 heavy atoms and 14 electrons) takes 3.63 ± 0.37 ms (average and standard deviation over 100 runs, respectively). On top of this, DSP takes 0.20 ± 0.04 ms, and CIS + DSP takes 11.36 ± 0.73 ms. Oscillator strengths were calculated with the dipole length approximation.^42,56,57^ Some compounds exhibit negative triplet excitation energies and, in some cases, even negative singlet excitation energies, which indicates a restricted–unrestricted instability of the ground-state wave function. We have here taken the pragmatic approach to ignore these issues, while we will highlight below some examples where it occurs and if it has any effect on the overall results. This approach can be partly justified as the reference data also do not include any stability analysis. The results should anyway be indicative of the gap between the lowest singlet and triplet states with open-shell character, regardless of whether they are lower in energy than the closed-shell singlet.

We used four separate data sets for this study. The first is a set of azaphenalenes previously studied by some of us, comprising 256 substituted compounds, for which excitation energies were computed with (second-order) algebraic-diagrammatic construction, ADC(2)/cc-pVDZ, and geometries were optimized with B3LYP/cc-pVDZ.^9^ The second is a set of 138 substituted azaazulenes with ADC(2)/cc-pVDZ excitation energies and optimized with the B97-3c composite method.^58^ The third is a set of 16 rationally designed scaffolds, which, including substitution, amounts to 68,695 unique compounds, optimized at the B97-3c level and with excitation energies at the ADC(2)/cc-pVDZ level.^59^ The fourth is a set of 315 nonalternant hydrocarbons including substitutions, divided into three subsets of size 76, 187, and 52, optimized at the ωB97X-D/def2-TZVP level and with excitation energies at the CC2/aug-cc-pVTZ level. Below, we compare our PPP-based method to the reference levels at the DFT-optimized geometries given in the data sets. For the azaphenalene data set, we also investigated the impact of geometry. An initial geometry of each molecule was generated with the EmbedMolecule function in RDKit^60^ and then optimized with MMFF94.^61^ This geometry was then further refined with either the GFN-FF force field,^62^ the GFN2-xTB semiempirical method,^63^ or the ANI-1ccx machine learning potential^64^ (as implemented in TorchANI^65^ and using ASE as the optimization backend^66^).

Data was handled with Pandas,^67^ and chemical structures were handled with the RDKit.^60^ Plots were generated with Matplotlib.^68^ Numerical calculations used NumPy^69^ and SciPy.^70^ Calculations and visualizations used Jupyter Notebooks,^71^ integrated into a Snakemake^72^ workflow for reproducible computation.

## Results and Discussion

### Orbital Decomposition of the Inverted Gap

**Figure 2:**
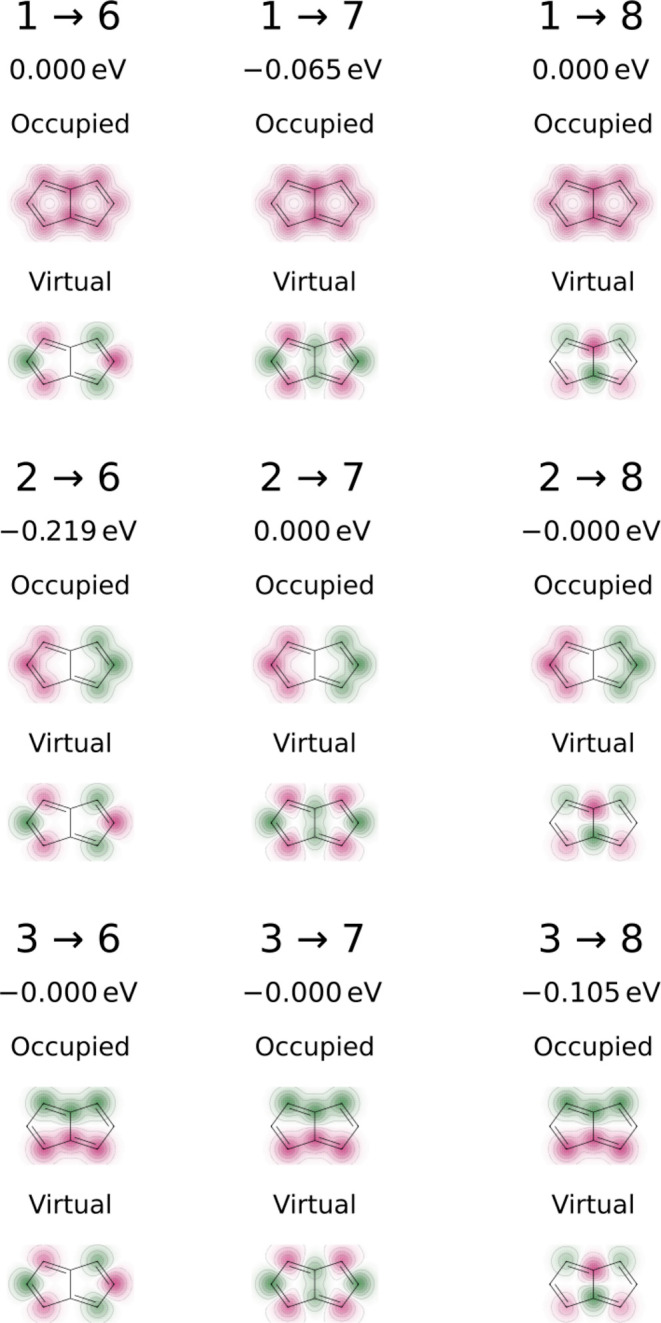
Excitations and their contribution to Δ*E*_SCF_^DSP^ = −0.389 eV for pentalene at the *D*_2*h*_ geometry and bond lengths of 1.4 Å.

We first demonstrate that our method is capable of capturing the inverted Δ*E*_ST_ for some of the model compounds. As shown by Toyota and co-workers, pentalene at the ideal *D*_2*h*_ geometry has an inverted gap, while relaxation to the minimum with *C*_2*h*_ symmetry (using B97-3c) leads to a normal gap.^5^ In our calculations, pentalene with equal bond lengths of 1.4 Å displays a small HOMO–LUMO overlap of 0.24, leading to a small exchange interaction of only 2*K* = 0.130 eV (Figure S2). The DSP correction Δ*E*_SCF_^DSP^ of −0.389 eV leads to a net predicted Δ*E*_ST,SCF_^DSP^ of −0.259 eV. Adding additional correlation with CIS leads to a Δ*E*_ST,CIS_^DSP^ of −0.177 eV. The simple perturbation theory model allows us to gain additional insights into the contributions to Δ*E*_SCF_^DSP^ as they correspond to single excitations from doubly occupied orbitals below the HOMO to unoccupied orbitals above the LUMO (Figure 2). Three excitations, HOMO – 3 → LUMO + 2, HOMO – 2 → LUMO + 1, and HOMO – 1 → LUMO + 3 (1 → 7, 2 → 6, and 3 → 8 in Figure 2) are identified as the main contributors to the spin polarization effect and could potentially be tuned by substituent effects.

**Figure 3:**
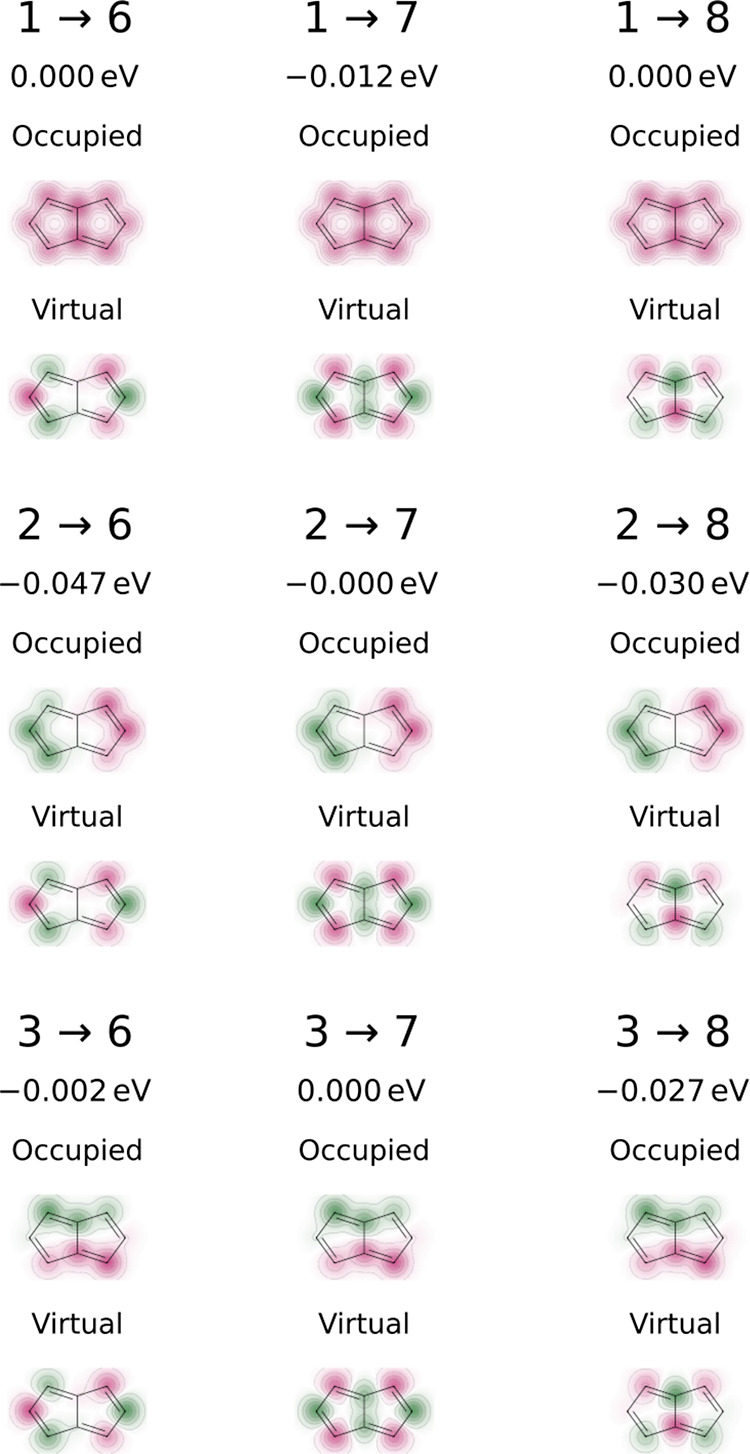
Excitations and their contribution to Δ*E*_SCF_^DSP^ = −0.118 eV for pentalene at the optimized *C*_2*h*_ geometry (optimized with B97-3c).

Using the relaxed *C*_2*h*_ geometry leads to dramatic changes in the frontier orbitals, with an increased HOMO–LUMO overlap of 0.86 and a sizable exchange interaction of 0.689 eV (Figure S3). Consequently, the calculated gap is now normal at Δ*E*_ST,SCF_^DSP^ = 0.570 eV, aggravated by a diminished Δ*E*_SCF_^DSP^ of only −0.118 eV. A similar result is obtained with CIS: Δ*E*_ST,CIS_^DSP^ = 0.914 eV. The reference value calculated with ADC(2) is 0.864 eV. The excitations contributing to Δ*E*_SCF_^DSP^ at the *D*_2*h*_ geometry have been significantly diminished, only partially alleviated by the addition of a minor stabilization from HOMO – 2 → LUMO + 3 (2 → 8 in Figure 3).

**Figure 4:**
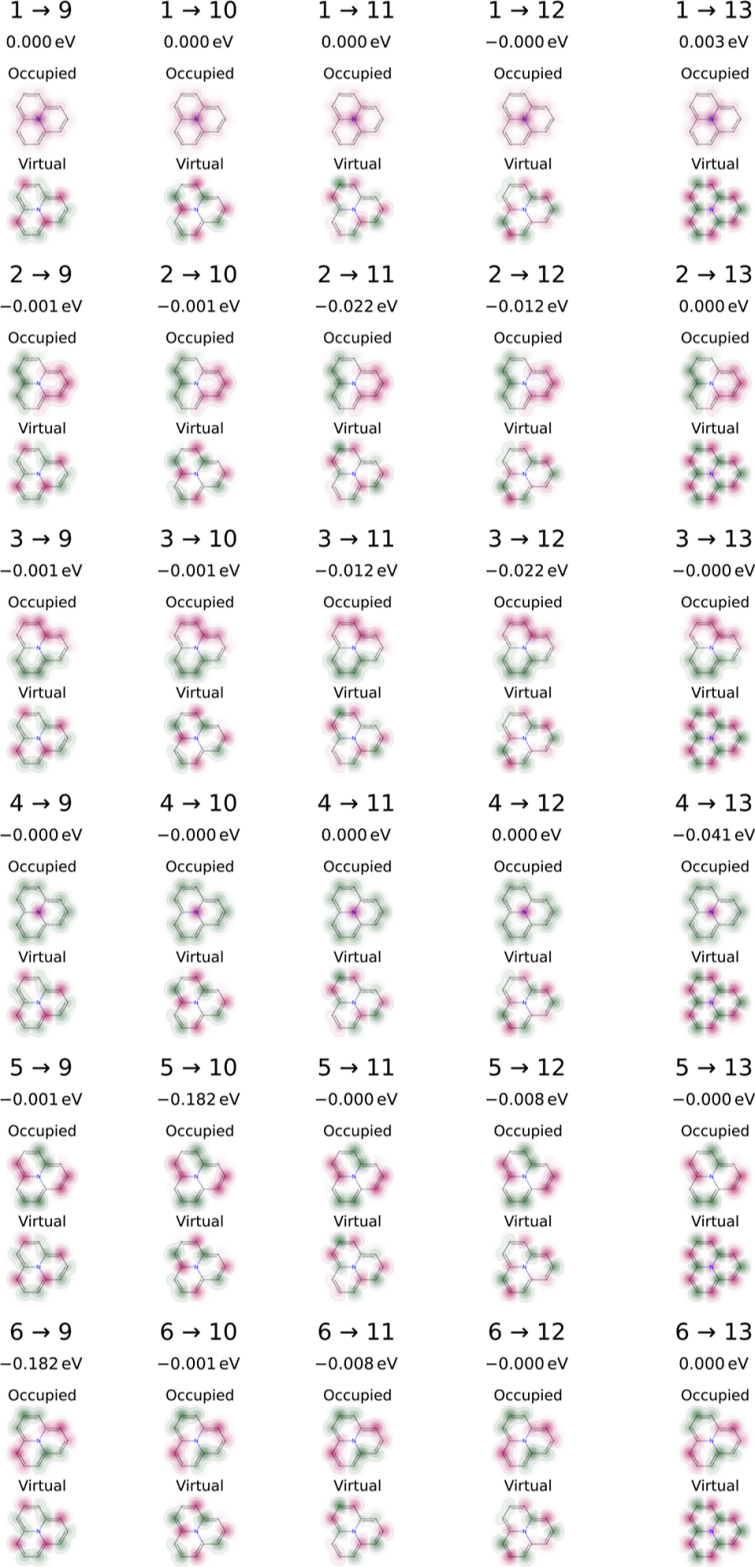
Excitations and their contribution to Δ*E*_SCF_^DSP^ = −0.492 eV for azaphenalene.

Azaphenalene is the prototypical molecule that started the renewed investigations into INVEST molecules^8,13^ and has been the subject of numerous studies with high-level quantum chemical methods. Can we capture the inverted gap, as first measured by Leupin and Wirz in 1980?^4^ The calculations reveal that the HOMO and LUMO are well separated with a spatial overlap of 0.14 and a correspondingly low exchange interaction of only 0.028 eV (Figure S4). The larger azaphenalene has 30 excitations that could possibly contribute to the large Δ*E*_SCF_^DSP^ of −0.492 eV, but the most important ones by far are those from the doubly degenerated HOMO – 1 and the doubly degenerated LUMO + 1 (5 → 10 and 6 → 9 in Figure 4). In total, Δ*E*_ST,SCF_^DSP^ = −0.464 eV, which is slightly smaller at the CIS level with Δ*E*_ST,CIS_^DSP^ = −0.321 eV.

To conclude this section on the model compounds, our PPP-based protocol is able to capture the inverted gap and also provides a qualitative understanding of the physical mechanism of DSP through identification and visualization of the corresponding excitations. For these two compounds, Δ*E*_ST_^DSP^ is more negative at the SCF level compared to CIS. Compared to ADC(2), it would seem that CIS is preferable, but to elucidate which method is better, we now turn to larger data sets.

### Local Chemical Space of Azaphenalenes and Azaazulenes

**Figure 5:**
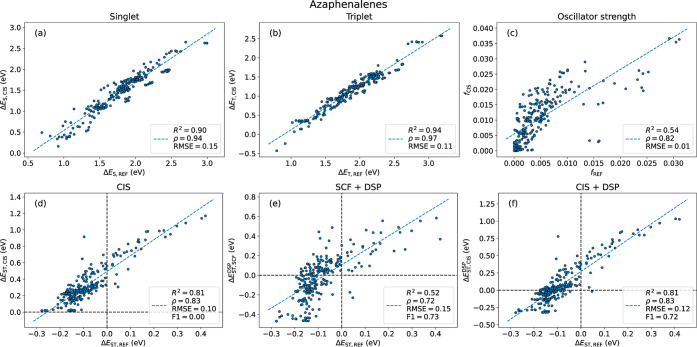
Excitation properties for azaphenalenes against the reference level. (a) Singlet excitation energies. (b) Triplet excitation energies. (c) Oscillator strengths. (d) Singlet–triplet energy gaps with CIS. (e) Singlet–triplet energy gaps with SCF + DSP. (f) Singlet–triplet energy gaps with CIS + DSP.

While our method captures the inverted gap of azaphenalene and pentalene, it also needs to capture trends with substitution to work effectively in virtual screening. We therefore calculated 256 substituted azaphenalenes that have previously been studied by some of us.^9^ The PPP-level S_1_ and T_1_ excitation energies are well correlated with those from ADC(2) with *R*^2^ values of 0.90 and 0.94, respectively (Figure 5a,b). Unfortunately, the oscillator strengths are not as well reproduced with an *R*^2^ of 0.54, although the Spearman ρ of 0.82 indicates that the values might be used to rank potential candidates (Figure 5c). The problem of obtaining accurate oscillator strengths at the PPP level of theory is well known in the literature and is especially exacerbated with low oscillator strengths which are prevalent in this data set.^73^ Crucially, Δ*E*_ST_ shows good correlations, with *R*^2^ = 0.81 using Δ*E*_ST,CIS_^DSP^ (Figure 5f). This *R*^2^ value is essentially unchanged from the gap at the CIS level without DSP (0.81, Figure 5d) and markedly better than that for the gap at the SCF level (0.52, Figure 5e), showing the importance of going beyond the SCF level to include at least some configuration interaction. The results are of similar or better quality recently achieved by Pernal and co-workers for a different set of azaphenalenes using SCF + DSP with orbitals from DFT.^52^ To further analyze the results in terms of a binary classification into normal/inverted, we calculated the true positives (TPs), true negatives (TNs), false positives (FPs), and false negatives (FNs), as well as a range of common classification scores (Table 1); for definitions, see eqs S1–S6. The most important metrics for virtual screening are, in our opinion, recall and specificity. The recall measures the fraction of inverted molecules that the protocol captures and is 0.58 at the SCF level and 0.56 at the CIS level. The specificity measures the fraction of noninverted molecules that the protocol identifies and is 0.94 at the SCF level and 0.97 at the CIS level. These metrics mean that we are able to capture a large proportion of the molecules with inverted gaps while filtering out most noninverted molecules. Below, we will show how we can improve the results even more with a linear correction to Δ*E*_ST,CIS_^DSP^ based on results from a wider set of compounds. The results for these azaphenalenes indicate that the PPP protocol could be used to prescreen candidates of this compound class for Δ*E*_ST_, while further pruning with another method is likely needed for oscillator strengths.

**Table 1:** Metrics for Azaphenalenes

	*R*^2^	ρ	RMSE	F1	ROC-AUC	accuracy	recall	specificity	TP	TN	FP	FN
SCF	0.52	0.72	0.15	0.73	0.76	0.62	0.58	0.94	128	32	2	94
CIS	0.81	0.83	0.12	0.72	0.77	0.62	0.56	0.97	125	33	1	97

**Figure 6:**
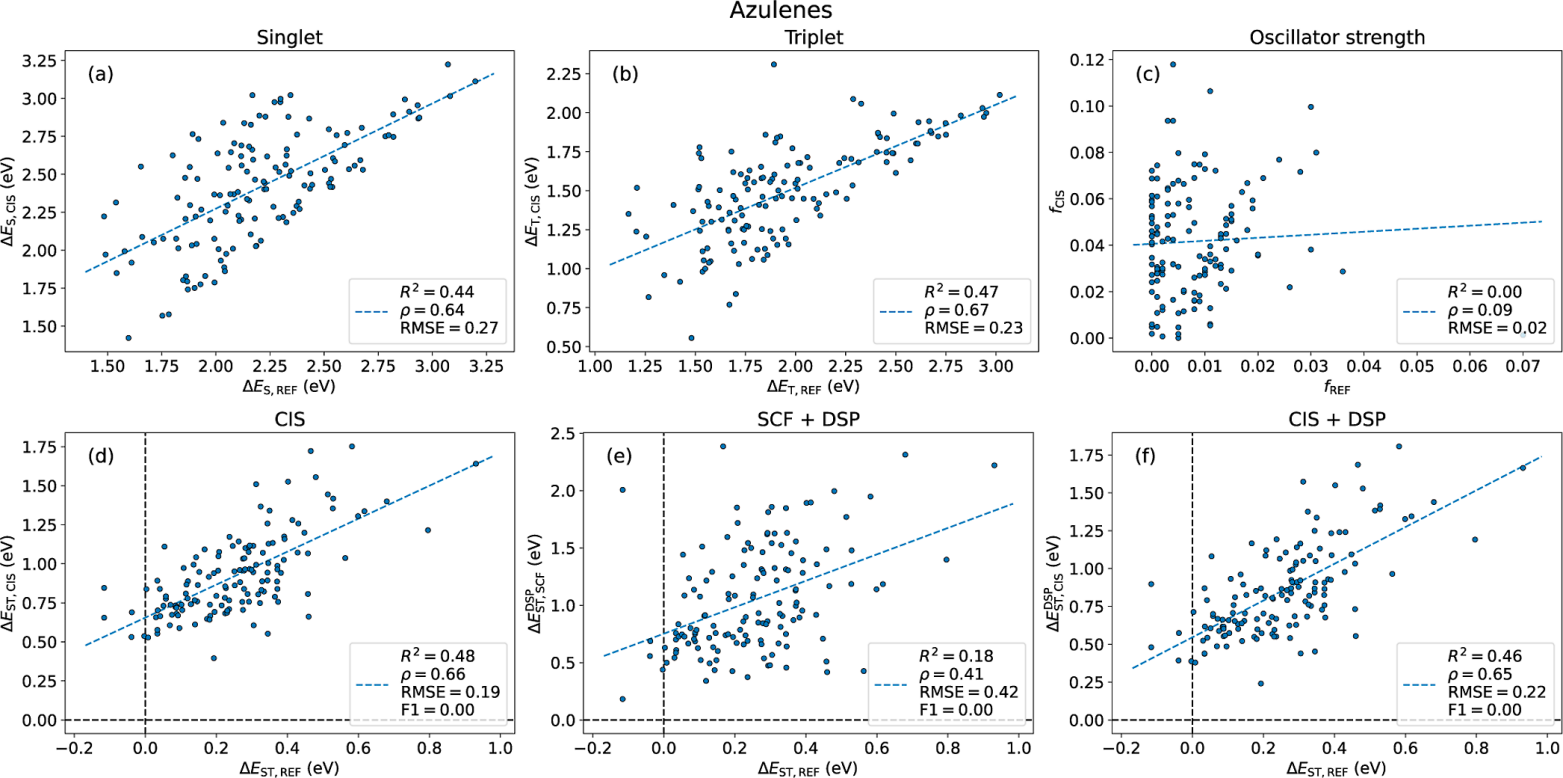
Excitation properties for azaazulenes against the reference level. (a) Singlet excitation energies. (b) Triplet excitation energies. (c) Oscillator strengths. (d) Singlet–triplet energy gaps with CIS. (e) Singlet–triplet energy gaps with SCF + DSP. (f) Singlet–triplet energy gaps with CIS + DSP.

While we see success for the azaphenalenes, the situation for the azaazulenes is unfortunately worse. The PPP level S_1_ and T_1_ excitation energies are only moderately correlated with those from ADC(2) with *R*^2^ values of 0.44 and 0.47, respectively (Figure 6a,b). For the oscillator strengths, the situation is rather catastrophic, with an *R*^2^ of 0.00 implying no correlation whatsoever (Figure 6c). The situation for the Δ*E*_ST_ is also worse than for the azaphenalenes, with *R*^2^ = 0.46 with CIS + DSP, although with a marked improvement over 0.18 with SCF + DSP (Figure 6e,f). Unfortunately, the negative gaps are not recovered, leading to recalls of 0.00, as none of the five inverted molecules could be identified (Table 2). It could be speculated that the worse performance for the azaazulenes comes from the fact that they are nonalternant molecules, for which the approximations are expected to be less applicable. Considering the contrasting performance for the azaphenalenes and azaazulenes, it seems to be clear that the PPP protocol will miss hits for some compound classes. To investigate the global performance of the model, we turned to a more diverse set with 16 different compound classes.

**Table 2:** Metrics for Azaazulenes

	*R*^2^	ρ	RMSE	F1	ROC-AUC	accuracy	recall	specificity	TP	TN	FP	FN
SCF	0.18	0.41	0.42	0.00	0.50	0.96	0.00	1.00	0	133	0	5
CIS	0.46	0.65	0.22	0.00	0.50	0.96	0.00	1.00	0	133	0	5

### Screening Widely

**Figure 7:**
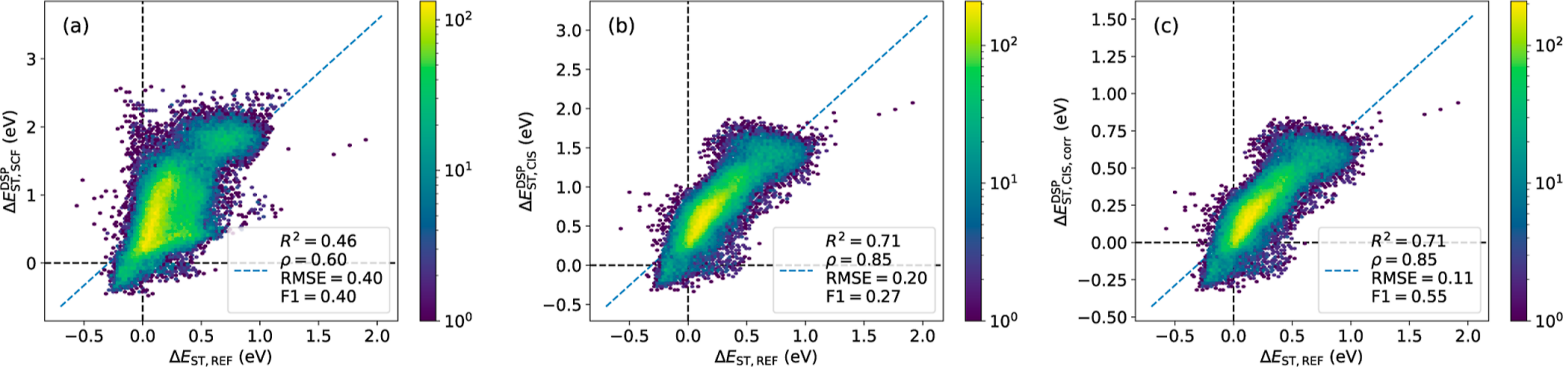
Singlet–triplet energy gaps for the rational design set against the reference level for (a) SCF + DSP, (b) CIS + DSP, and (c) CIS + DSP and linear correction.

In this section, we used a data set previously generated by some of us using rational design rules, comprising 16 different molecular scaffolds, the details of which will be presented elsewhere. Out of 68,695 compounds, only 5 cyclobutadienes failed to compute as they had rearranged in the DFT simulations, and our topology detection algorithm interpreted them as having formed a transannular single bond. This corresponds to a success rate of 99.99%. To put this into context, ∼25% of the CASSCF calculations and 9% of the CIS calculations failed in a recent HTVS workflow by Padula and co-workers.^17^ The total runtime using four cores on the M2 processor was 1.82 CPU hours, with a mean time per compound of 0.09 s. The S_1_ and T_1_ excitation energies are fairly reproduced with an *R*^2^ of 0.66 and 0.70 against ADC(2), respectively (Figure S5a,b). As seen in Figure 7a, the Δ*E*_ST_ at the SCF + DSP level shows a moderate *R*^2^ of 0.46, which increases significantly to 0.71 at the CIS + DSP level (Figure 7b). However, due to a systematic overestimation of Δ*E*_ST_, the F1 score is still low at 0.27 and the recall is only 0.16 (Table 3). We therefore added a linear correction Δ*E*_ST,CIS,LC_^DSP^ = 0.53 × Δ*E*_ST,CIS_^DSP^ −0.15, which increases the F1 score to 0.55 and the recall to 0.51 (Figure 7c). The specificity simultaneously decreases from 0.99 to 0.96, but overall, the linear correction would be preferable for virtual screening where there is a strong focus on finding rare hits. Applying the linear correction to the azaphenalenes (Figure S10a) and azaazulenes (Figure S10b) leads to an improvement in the recall of the former from 0.56 to 0.94 while still none of the inverted azaazulenes are recovered. The oscillator strengths are unfortunately poorly correlated for the rational design set with *R*^2^ = 0.21, although the Spearman rank correlation is more encouraging at 0.58 (Figure S5c). Taken together, the results reinforce the conclusions from the case study of the azaphenalenes that the PPP protocol is suitable for screening Δ*E*_ST_ while it struggles for oscillator strengths.

**Table 3:** Metrics for a Rationally Designed Set

	*R*^2^	ρ	RMSE	F1	ROC-AUC	accuracy	recall	specificity	TP	TN	FP	FN
SCF + DSP	0.46	0.60	0.40	0.40	0.63	0.91	0.27	0.99	2073	60,490	561	5566
CIS + DSP	0.71	0.85	0.20	0.27	0.58	0.90	0.16	0.99	1227	60,736	315	6412
CIS + DSP + LC	0.71	0.85	0.11	0.55	0.73	0.91	0.51	0.96	3867	58,592	2459	3772

A more detailed breakdown of the correlations among the different scaffolds (Figure S6) shows that the highest *R*^2^ of 0.82 is obtained for dicyclopenta[a,e]cyclooctene, while the lowest is obtained for bowtiene with 0.07. For recall, the corresponding range is 0.92 for zurlene to 0.01 for phenazulene. The wide range in *R*^2^ and recall further reinforces that the method struggles with some particular compound classes despite its favorable global performance. Also, there seems to be no clear performance difference between alternant and nonalternant molecules, as we speculated above based on the results for azaphenalenes and azaazulenes. Despite these problems, the method is able to capture inverted molecules in 15 out of the 15 scaffolds where they occur. Even for the azulenes included in this screening set, at least some candidates are found, in contrast to the findings above for the azaazulenes. The per-scaffold oscillator strengths are arguably sufficiently good to allow for local screening in some cases (Figure S9). The per-scaffold S_1_ and T_1_ excitation energies can be found in Figures S7 and S8, respectively. Notable failures are seen for cyclobuta-1,3-diene with *R*^2^ values of 0.29 and 0.27, respectively. Additionally, phenazulene and dicyclopenta[*a*,*c*]cyclooctene show some negative T_1_ excitation energies, indicating restricted–unrestricted instabilities. A closer inspection of the data set reveals that 145 out of 68,690 calculated compounds show negative triplet excitation energies (0.21%, see Table S4). This can be compared to 0.78% for the azaphenalenes and 0.00% for the azulenes (Figure S11 and Table S4). Broken down over scaffolds, the results indicate that some of the outliers in the calculated Δ*E*_ST_ might be explained this way (Figure S12), although the absolute numbers are very small compared with the total number of compounds.

### External Validation

To further test the validity of the method, we calculated a series of compounds recently published by Garner et al.^18^ The data set comes in three parts: (1) nonalternants, (2) nonalternants with constrained high-symmetry geometries (we here follow the original terminology and call these “avoided symmetry”), and (3) substituted nonalternants (here called “substituted”). Out of the substituted compounds, four could not be calculated, as they contain four-coordinate P atoms for which our PPP model lacks parameters. We here opted to use the CC2/aug-cc-pVDZ data from the original paper as the reference as it is the highest level which has the most complete coverage of the data set.

**Figure 8:**
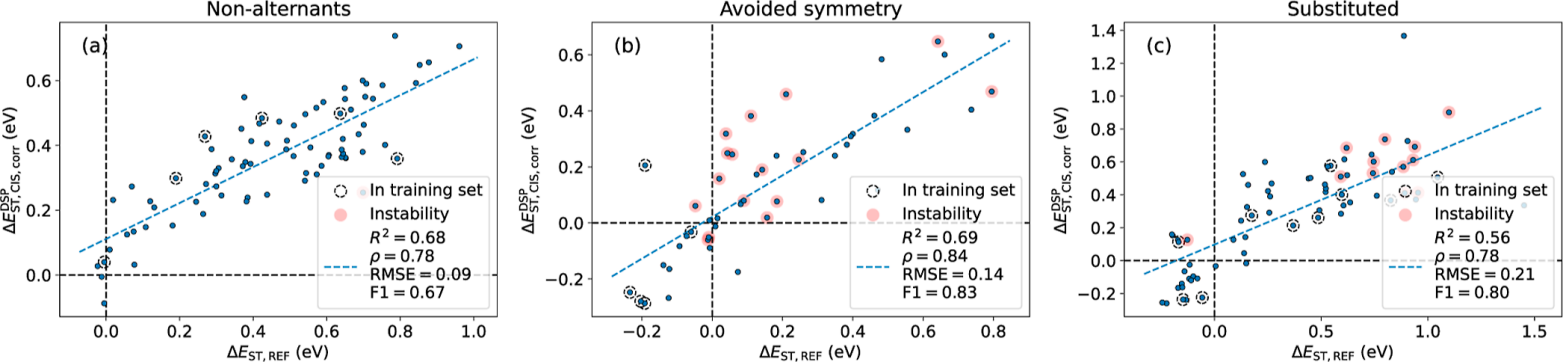
Singlet–triplet energy gaps for CIS + DSP and linear correction against the reference level for the subsets of (a) nonalternants, (b) nonalternants constrained to higher symmetry, and (c) substituted nonalternants. Compounds contained in the rationally designed sets are marked with dashed circles and those with negative triplet excitation energies are marked with a semitransparent red circle.

Our computed Δ*E*_ST_ with the linearly corrected CIS + DSP shows fair correlations with the CC2 values, with *R*^2^ values of 0.68, 0.69, and 0.56, respectively (Figure 8). Gratifyingly, the recalls are 0.50, 0.80, and 0.74, respectively, showing that we can recover a large part of the inverted molecules found with the much more expensive CC2 method (Table 4). Although some of the compounds are also present in the rationally designed set that we used for the linear correction above, we believe that they are not sufficiently many to compromise the use of the Garner data for external validation (Figure 8). We also indicate in the plots which compounds show instabilities (Figure 8). There seems to be no clear deterioration in the performance, although the reference data might also exhibit instabilities as there is no mention of any stability analysis in the original manuscript.^18^

**Table 4:** Metrics for the Data Set from Garner et al.

	*R*^2^	ρ	RMSE	F1	ROC-AUC	accuracy	recall	specificity	TP	TN	FP	FN
nonalternants	0.68	0.78	0.09	0.67	0.75	0.97	0.50	1.00	2	75	0	2
avoided symmetry	0.69	0.84	0.14	0.83	0.87	0.89	0.80	0.94	12	30	2	3
substituted	0.56	0.78	0.21	0.80	0.85	0.90	0.74	0.96	14	50	2	5

In summary, the external validation shows that the PPP method can recover inverted molecules at a fraction of the cost of more expensive methods, such as CC2.

### Effect of Geometry

Even though our PPP protocol is very fast, this speed would not be beneficial if DFT-optimized geometries were required to accurately reproduce the energy gaps. In the benchmarking above, we used the same geometries as in the original data sets to allow for a comparison based on equal footing. For ultrafast screening, we would need geometries from force fields, semiempirical methods, or machine learning potentials that can be obtained on a similar time scale as the PPP results. We chose the azaphenalene data set for a limited benchmark and optimized the geometries with the MMFF94 (average runtime of 11.45 ms) and GFN-FF (40.18 ms) methods, the GFN2-xTB semiempirical method (228.13 ms), and the ANI-1ccx machine learning potential (572.83 ms, CPU-based). While the two force-field methods are insufficiently accurate, both GFN2-xTB and ANI-1ccx provide sufficiently good geometries for screening (Figure S13, Figure S14, and Table S3). In particular, GFN2-xTB shows a good correlation (*R*^2^ = 0.94) with the gaps in the DFT-optimized geometries. Compared to the reference ADC(2)/cc-pVDZ level, the results with GFN2-xTB geometries are equally good to those from DFT geometries (*R*^2^ of 0.80 vs 0.81 and recall of 0.57 vs 0.56, respectively). The optimization runtime of 228.13 ms is slower, but of a similar magnitude compared to the runtime of the PPP protocol itself (47.05 ms), and significantly faster than DFT optimization. Based on the good performance, it is likely that the GFN2-xTB geometries could be used in virtual screening campaigns.

## Conclusions and Outlook

To conclude, we have shown that the PPP theory, a simple semiempirical π-electron theory with a minimal valence basis, can be used to screen for molecules with inverted singlet–triplet energy gaps, both locally and globally. Unfortunately, the method does not seem capable of screening for oscillator strengths with the same accuracy, with exceptions for some scaffolds. The chief limitation of the method is that it only includes the π-electron system and therefore struggles with (1) functional groups without a clear σ–π separation (found in common functional groups such as sulfonyls), (2) inductive effects, and (3) neglect of *n* → π* transitions. Further limitations of the approach used in this study include (4) the lack of a solvation model and need for (5) already optimized geometries. We believe that at least some of these limitations could be mitigated by a reparametrization specifically targeting inverted gaps, while our preliminary tests indicate that geometries from the fast GFN-xTB family of methods would be suitable. Alternatively, these limitations could be overcome by applying the perturbation theory description of DSP^3^ to the more costly but potentially more accurate all-electron semiempirical methods, such as those of the OMx family.^74^

Taken together, we foresee that the presented methodology can be used for ultrahigh-throughput virtual screening campaigns and in inverse design algorithms, followed by curation of hits using more accurate quantum chemical methods. Active learning schemes could also be used with machine learning corrections to the PPP singlet–triplet gaps. The method has great potential to accelerate the discovery of the next generation of OLED materials based on INVEST.

## Data Availability

The codes to perform all calculations and to generate the PDF of the manuscript using the Quarto publishing system are found at https://github.com/kjelljorner/ppp-invest. Snakemake^72^ was used as the workflow manager, ensuring reproducible data generation. An archive of the workflow run used to generate this manuscript can be found on 10.5281/zenodo.10569815 together with all output data generated.

## Supporting Information Available

Definition of classification metrics, semiempirical parameters, and additional figures and tables (PDF)
