# Supplementary material for: Ultrafast Computational Screening of Molecules with Inverted Singlet–Triplet Energy Gaps Using the Pariser–Parr–Pople Semiempirical Quantum Chemistry Method
Source: J Phys Chem A. 2024 Mar 14;128(12):2445–56. doi: 10.1021/acs.jpca.3c06357 (PMC10983003; doi:10.1021/acs.jpca.3c06357)
Supplement: Supplementary file 1 — jp3c06357_si_001.pdf [file jp3c06357_si_001.pdf]

Ultrafast Computational Screening of Molecules with Inverted Singlet-triplet Energy Gaps Using the Pariser-Parr-Pople Semi-empirical Quantum Chemistry Method

Kjell Jorner[a,b,c,d,*], Robert Pollice[e,c,d,*], Cyrille Lavigne[c,d], Alán Aspuru-Guzik[c,d,f,g,h,j,i,*]

[a] *ETH Zürich, Institute of Chemical and Bioengineering, Department of Chemistry and Applied Biosciences, Vladimir-Prelog-Weg 1, Zürich, Switzerland, CH-8093*

[b] *Chalmers University of Technology, Department of Chemistry and Chemical Engineering, Kemigården 4, Gothenburg, Sweden, SE-41258*

[c] *University of Toronto, Chemical Physics Theory Group, Department of Chemistry, 80 St. George St., Toronto, Canada, M5S 3H6*

[d] *University of Toronto, Department of Computer Science, 40 St. George St., Toronto, Canada, M5S 2E4*

[e] *University of Groningen, Stratingh Institute for Chemistry, Nijenborgh 4, Groningen, The Netherlands, 9747 AG*

[f] *University of Toronto, Department of Chemical Engineering & Applied Chemistry, 200 College St., Toronto, Canada, M5S 3E5*

[g] *University of Toronto, Department of Materials Science & Engineering, 184 College St., Toronto, Canada, M5S 3E4*

[h] *Vector Institute for Artificial Intelligence, 661 University Ave. Suite 710, Toronto, Canada, M5G 1M1*

[i] *Lebovic Fellow, Canadian Institute for Advanced Research (CIFAR), 661 University Ave., Toronto, Canada, M5G 1M1*

[j] *Acceleration Consortium, University of Toronto, 700 University Ave., Toronto, Canada, M5G 1Z5*

Supporting Information

---

*Corresponding author

Email addresses: kjell.jorner@chem.ethz.ch (Kjell Jorner), r.pollice@rug.nl (Robert Pollice), aspuru@utoronto.ca (Alán Aspuru-Guzik)

Metrics

For definitions, see the review by Tharwat.[S1] The F1 score is the harmonic mean of the precision and sensitivity, and is defined as

$$\frac{2\mathrm{TP}}{2\mathrm{TP} + \mathrm{FP} + \mathrm{FN}} \tag{S1}$$

The accuracy is defined as

$$\frac{\mathrm{TP} + \mathrm{TN}}{\mathrm{TP} + \mathrm{TN} + \mathrm{FP} + \mathrm{FN}} \tag{S2}$$

The specificity, or true negative rate (TNR), is defined as

$$\frac{\mathrm{TN}}{\mathrm{TN} + \mathrm{FP}} \tag{S3}$$

The recall, or true positive rate (TPR), is defined as

$$\frac{\mathrm{TP}}{\mathrm{TP} + \mathrm{FN}} \tag{S4}$$

The fallout, or false positive rate (FPR), is defined as

$$\frac{\mathrm{FP}}{\mathrm{FP} + \mathrm{TN}} \tag{S5}$$

The ROC-AUC, or the area under the so-called receiver operating characteristic (ROC) curve, is given as

$$\int_{x=0}^{1} \mathrm{TPR}(\mathrm{FPR}^{-1}(x))dx \tag{S6}$$

i.e., the area under the curve with the FPR on the x axis and TPR on the y axis.

Table S1: Valence state ionization potentials and electron affinities for the elements in the dataset. For elements that can contribute more than one π-electron, X1 is the element X with one π-electron, X2 with two π-electrons.

Atom type	IP (eV)	EA (eV)
C	11.16	0.03
N1	14.12	1.78
N2	28.71	11.96
O1	17.70	2.47
O2	34.08	15.30
P1	11.64	1.80

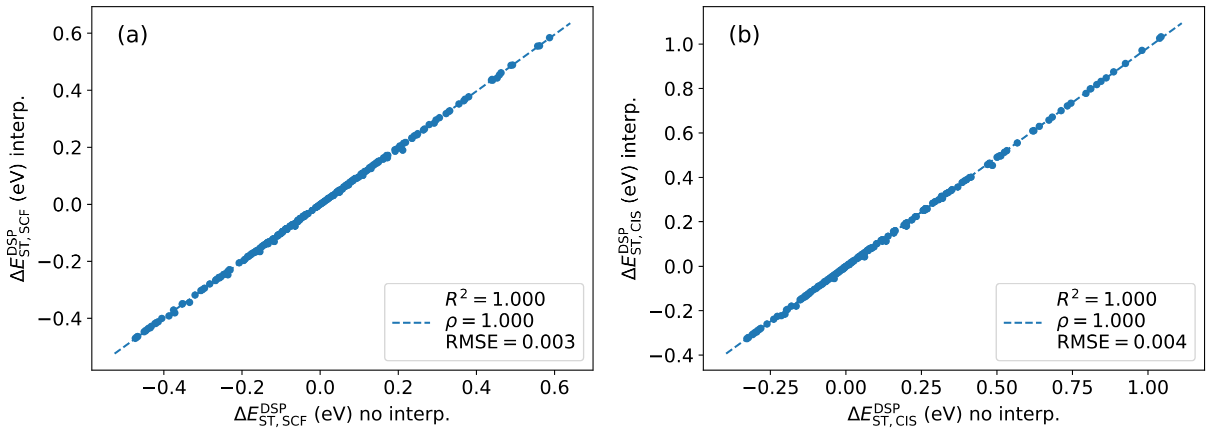

Figure S1: Comparison of singlet-triplet energy gaps using non-interpolated and interpolated overlap integrals for (a) SCF + DSP (b) CIS + DSP.

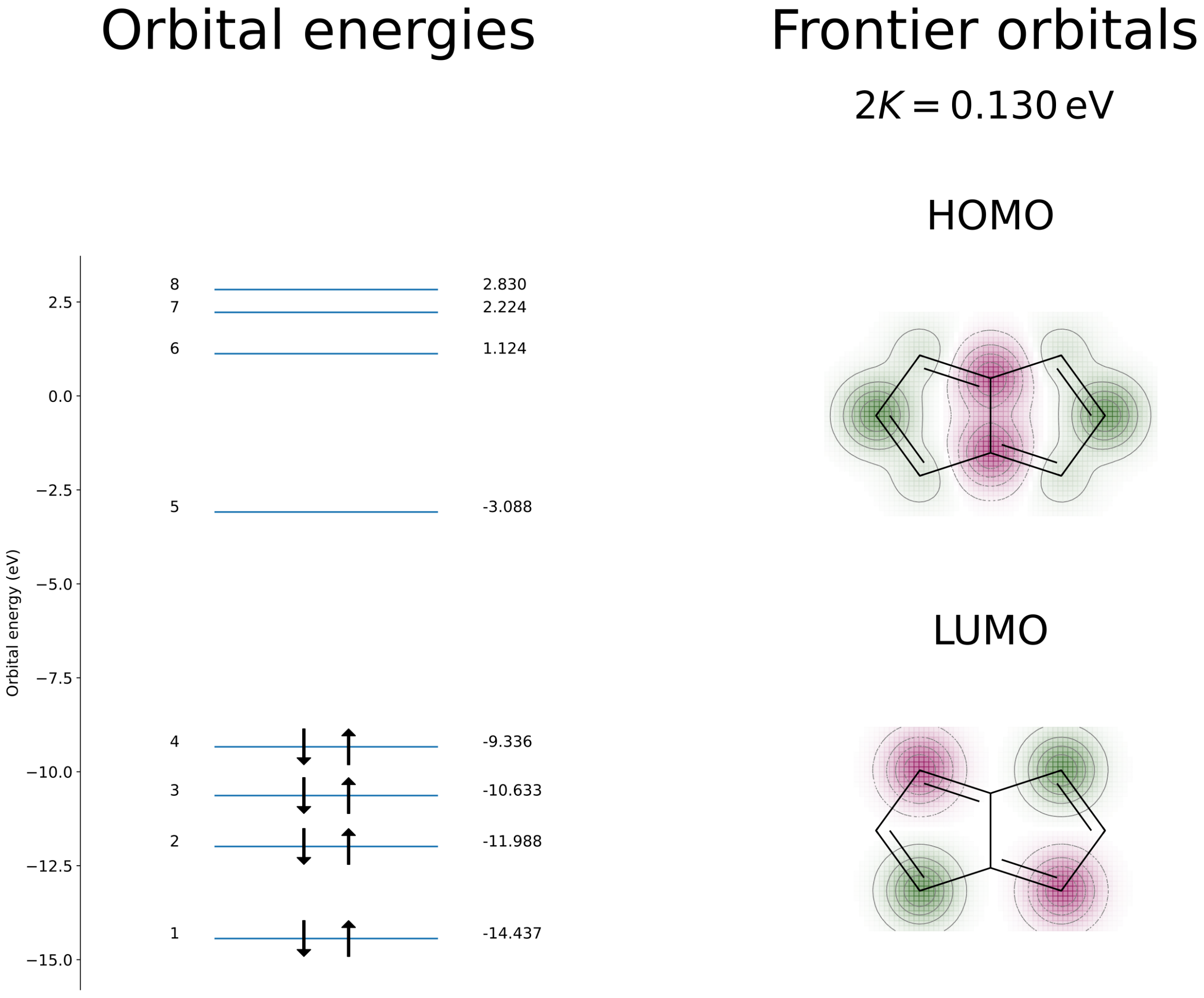

Figure S2: Orbital energies and frontier orbitals of pentalene at the idealized $D_{2\mathrm{h}}$ geometry and bond lengths of 1.4 Å.

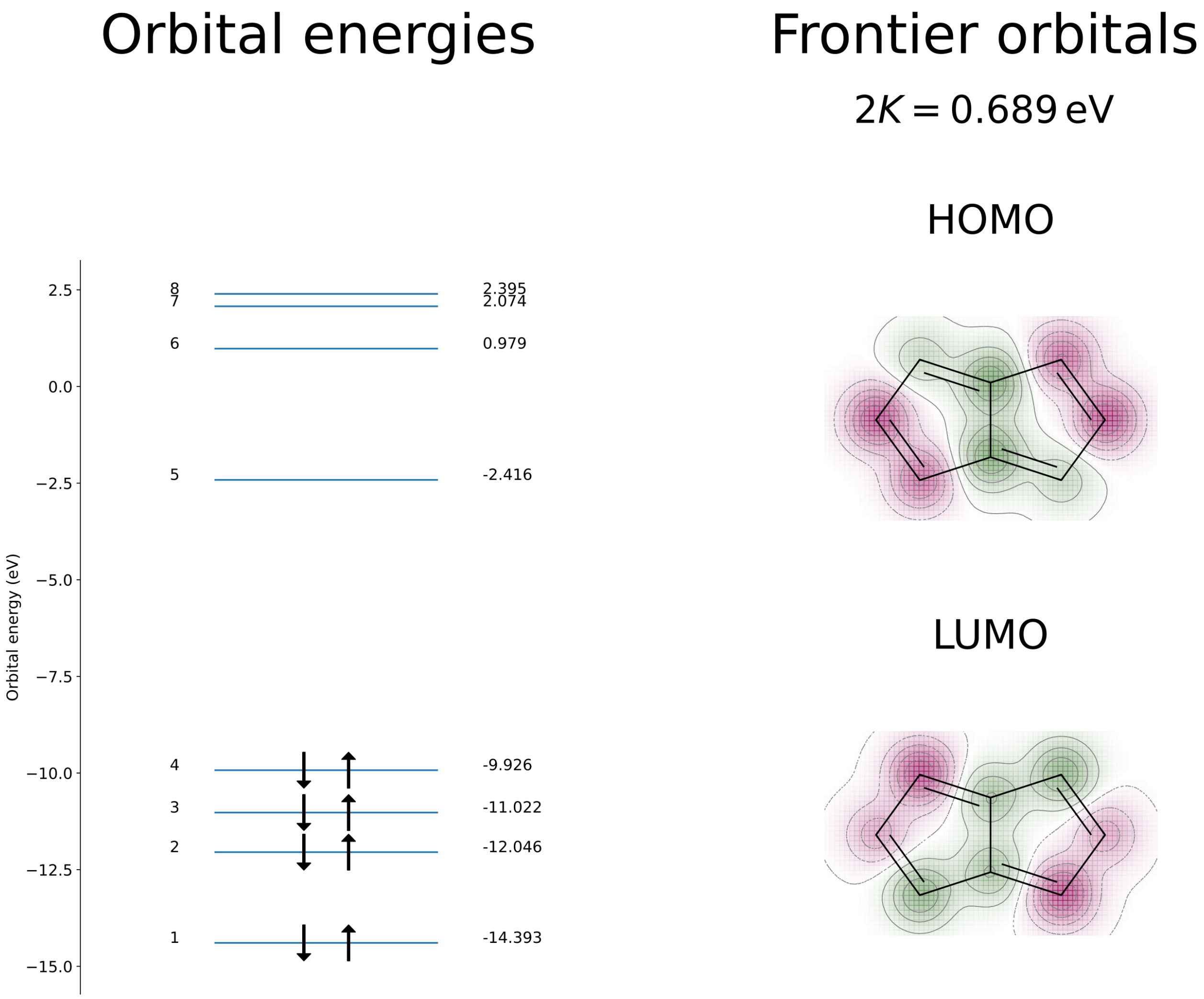

Figure S3: Orbital energies and frontier orbitals of pentalene at the optimized C_{2h} geometry (optimized with B97-3c).

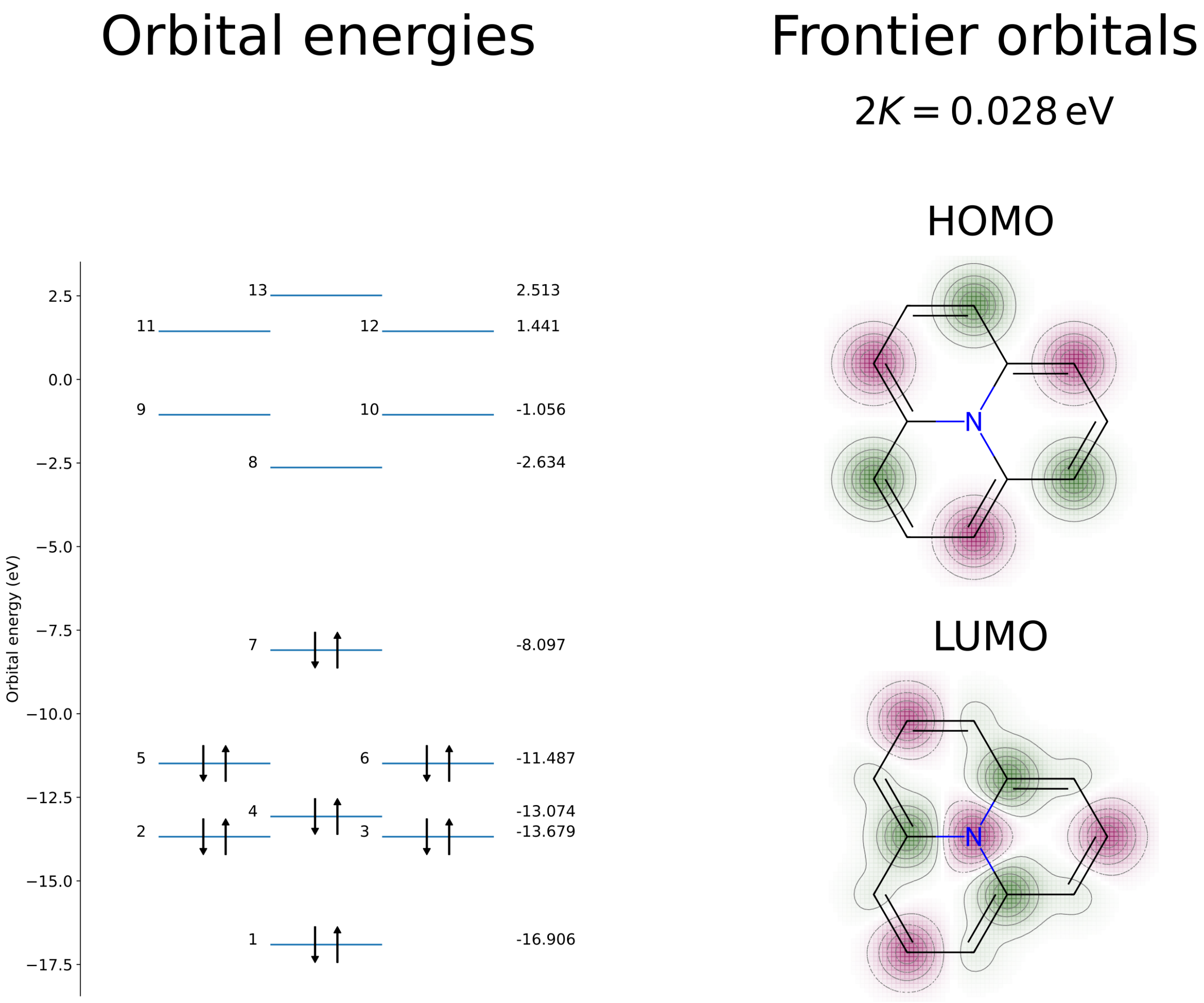

Figure S4: Orbital energies and frontier orbitals of azaphenalene.

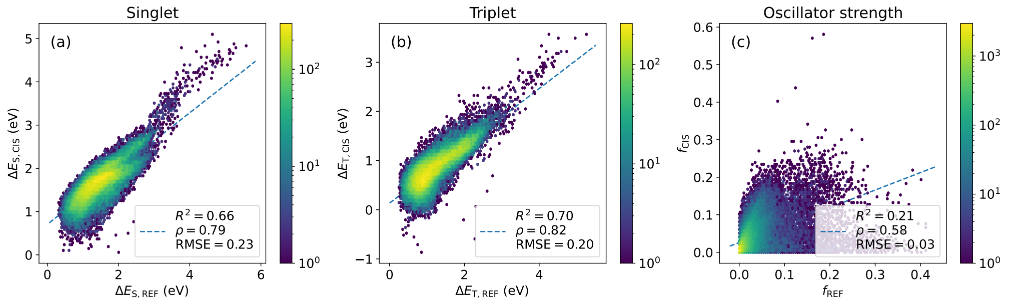

Figure S5: Singlet excitation energies, triplet excitation energies and oscillator strengths for the rationally designed dataset against the reference level.

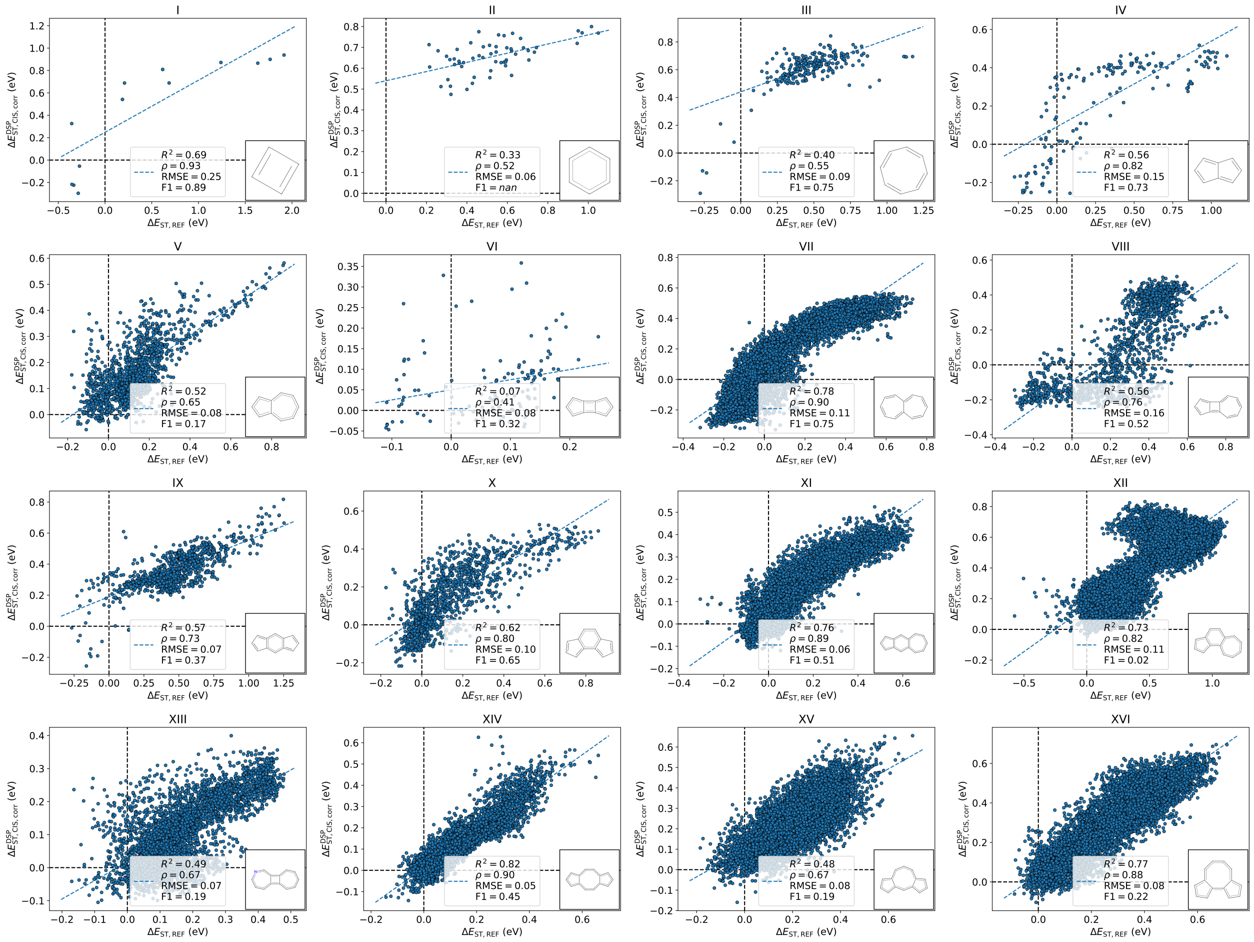

Figure S6: Singlet-triplet energy gaps split by scaffold for the rationally designed dataset against the reference level.

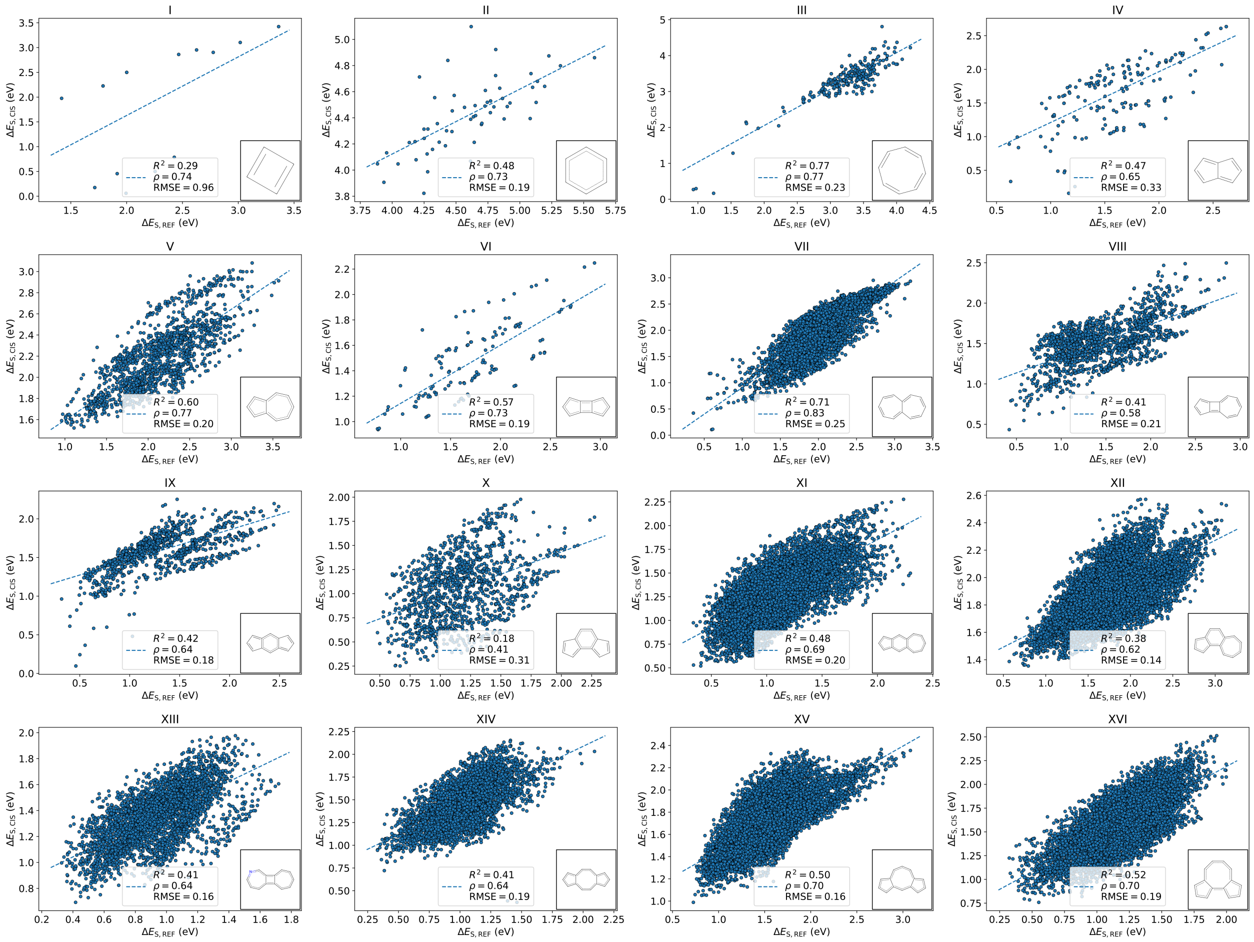

Figure S7: Singlet excitation energies split by scaffold for the rationally designed dataset against the reference level.

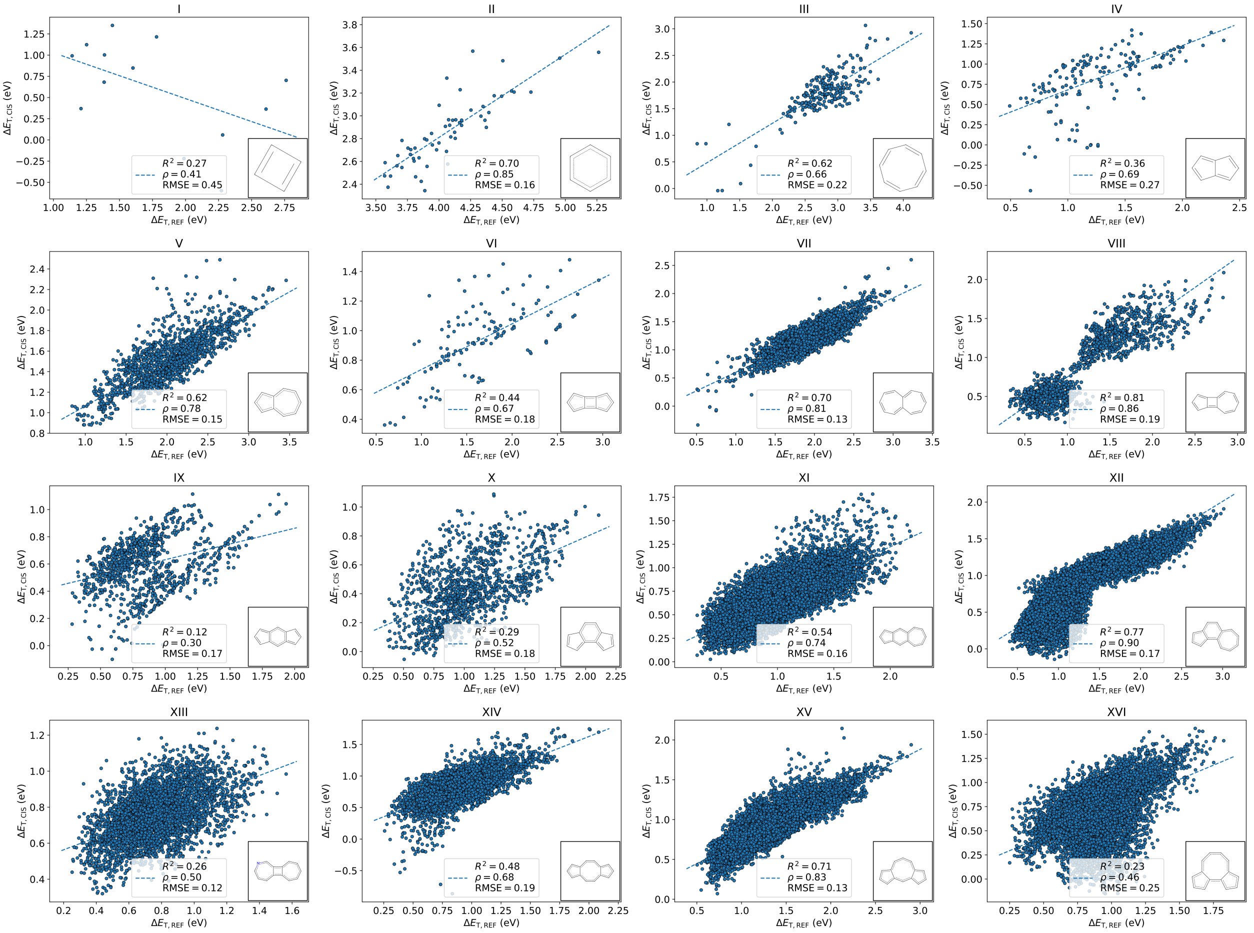

Figure S8: Triplet excitation energies split by scaffold for the rationally designed dataset against the reference level.

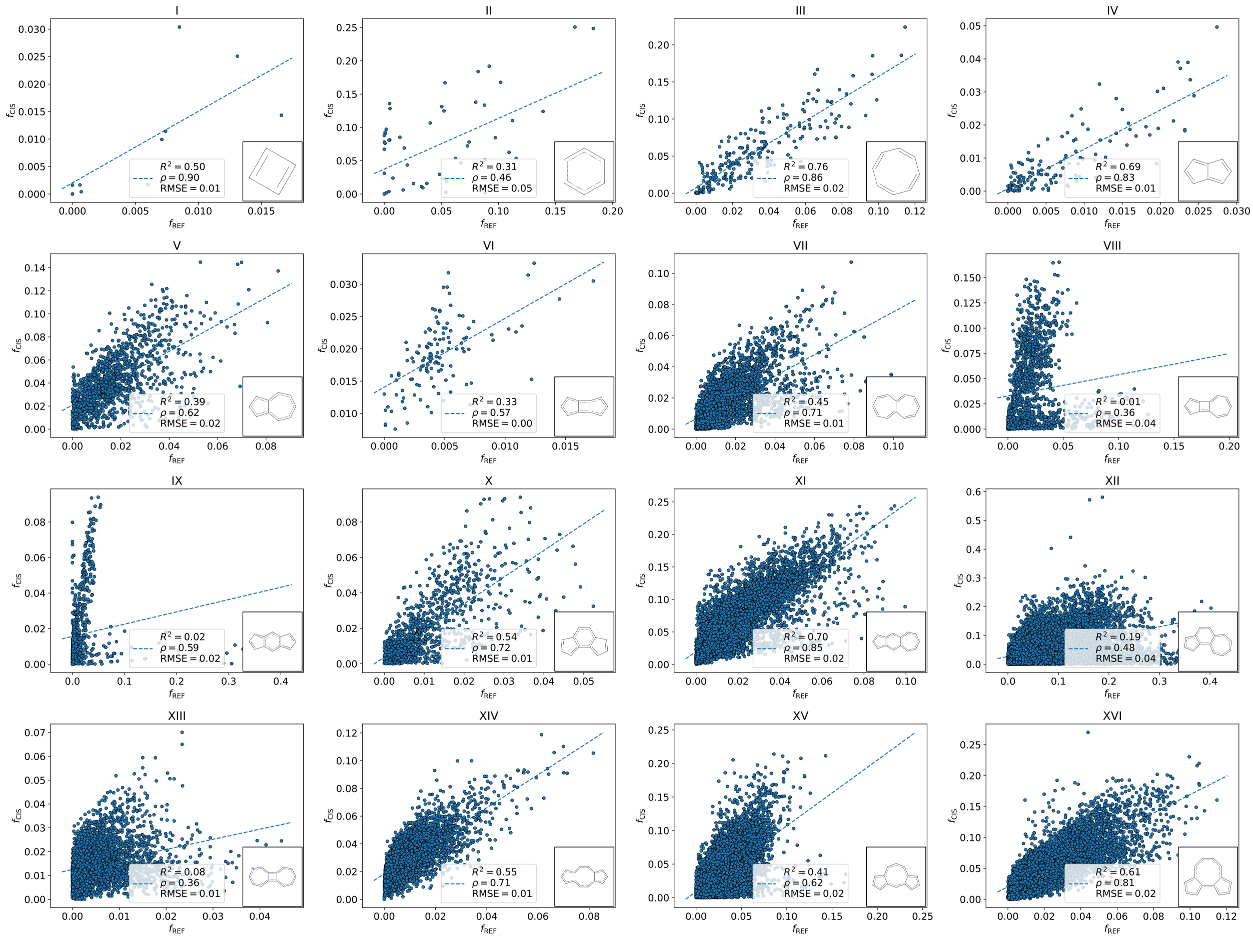

Figure S9: Oscillator strengths split by scaffold for the rationally designed dataset against the reference level.

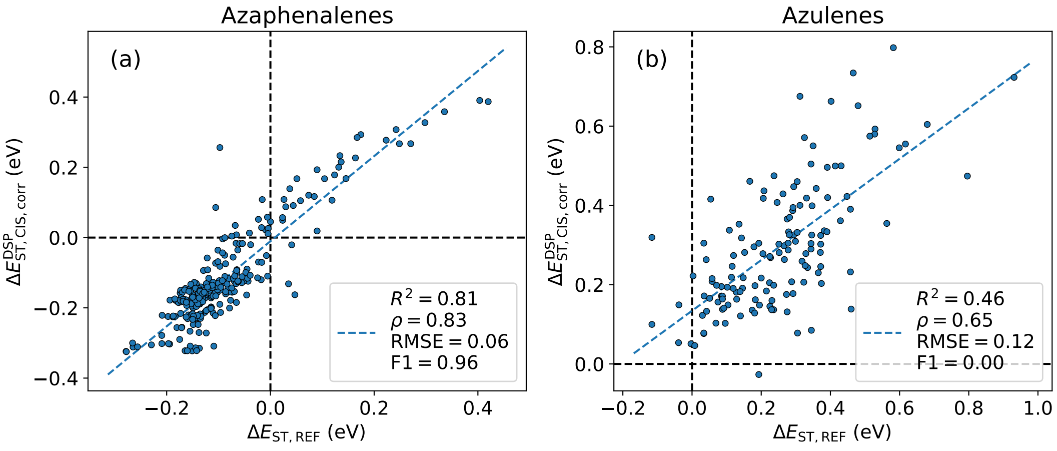

Figure S10: Singlet-triplet energy gaps with CIS + DSP and linear correction for the (a) azaphenalenes and (b) azaazulenes against the reference level.

Table S2: Metrics split by scaffold for the rationally designed dataset.

	R^2	ρ	RMSE	F1	ROC-AUC	Accuracy	Recall	Specificity	TP	TN	FP	FN
I	0.69	0.93	0.25	0.89	0.90	0.93	0.80	1.00	4	10	0	1
II	0.33	0.52	0.06	-	-	1.00	-	1.00	0	56	0	0
III	0.40	0.55	0.09	0.75	0.80	0.99	0.60	1.00	3	331	0	2
IV	0.56	0.82	0.15	0.73	0.81	0.88	0.66	0.95	39	178	9	20
V	0.52	0.65	0.08	0.17	0.54	0.83	0.10	0.99	27	1208	13	245
VI	0.07	0.41	0.08	0.32	0.56	0.70	0.32	0.80	9	78	19	19
VII	0.78	0.90	0.11	0.75	0.80	0.83	0.67	0.93	2175	4860	392	1085
VIII	0.56	0.76	0.16	0.52	0.80	0.72	0.92	0.69	388	1470	673	33
IX	0.57	0.73	0.07	0.37	0.62	0.98	0.24	1.00	9	1644	2	29
X	0.62	0.80	0.10	0.65	0.76	0.82	0.62	0.90	215	841	97	134
XI	0.76	0.89	0.06	0.51	0.69	0.87	0.42	0.95	714	8436	417	972
XII	0.73	0.82	0.11	0.02	0.51	0.97	0.01	1.00	5	15085	17	439
XIII	0.49	0.67	0.07	0.19	0.61	0.84	0.35	0.87	72	3199	472	135
XIV	0.82	0.90	0.05	0.45	0.70	0.93	0.44	0.96	116	3556	140	149
XV	0.48	0.67	0.08	0.19	0.56	0.96	0.12	1.00	47	10263	44	353
XVI	0.77	0.88	0.08	0.22	0.60	0.96	0.22	0.98	44	7377	164	156

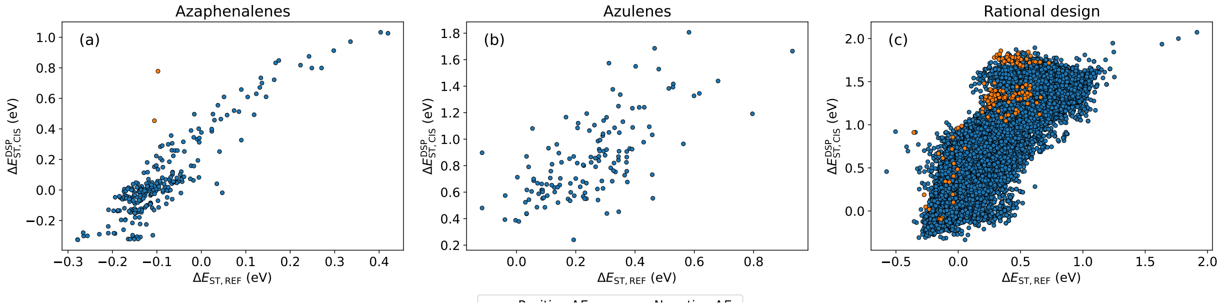

Figure S11: Singlet-triplet energy gaps with CIS + DSP against the reference level with points with negative triplet excitation energies marked for the (a) azaphenalenes and (b) azaazulenes (c) rationally designed datasets.

Table S3: Metrics for azaphenalenes at different geometries.

	R^2	ρ	RMSE	F1	ROC-AUC	Accuracy	Recall	Specificity	TP	TN	FP	FN
MMFF	0.25	0.45	0.19	0.00	0.50	0.13	0.00	1.00	0	34	0	222
GFNFF	0.29	0.58	0.12	0.78	0.69	0.68	0.67	0.71	149	24	10	73
GFN2-xTB	0.80	0.80	0.10	0.72	0.76	0.62	0.57	0.94	127	32	2	95
ANI-1ccx	0.74	0.74	0.11	0.77	0.81	0.67	0.62	1.00	138	34	0	84
DFT	0.81	0.83	0.12	0.72	0.77	0.62	0.56	0.97	125	33	1	97

Table S4: Statistics of compounds with negative triplet excitation energies.

	N(neg. triplet)	N(neg. singlet)	N(total)	%(neg. triplet)	%(neg. singlet)
Azaphenalenes	2	0	256	0.78	0.00

	N(neg. triplet)	N(neg. singlet)	N(total)	%(neg. triplet)	%(neg. singlet)
Azulenes	0	0	138	0.00	0.00
Rational design	145	0	68690	0.21	0.00

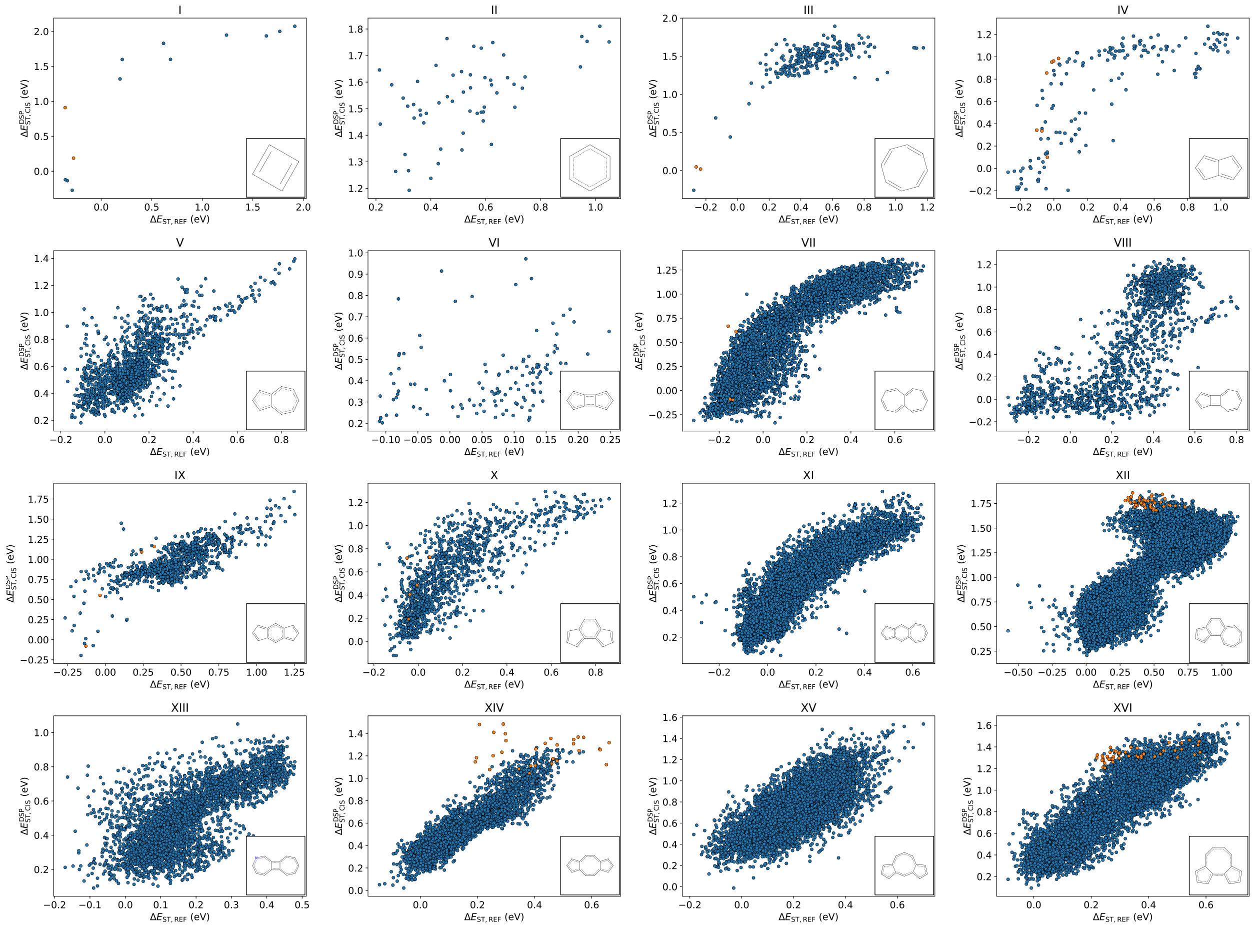

Figure S12: Singlet-triplet energy gaps with CIS + DSP against the reference level with points with negative triplet excitation energies marked split by scaffold for the rationally designed dataset.

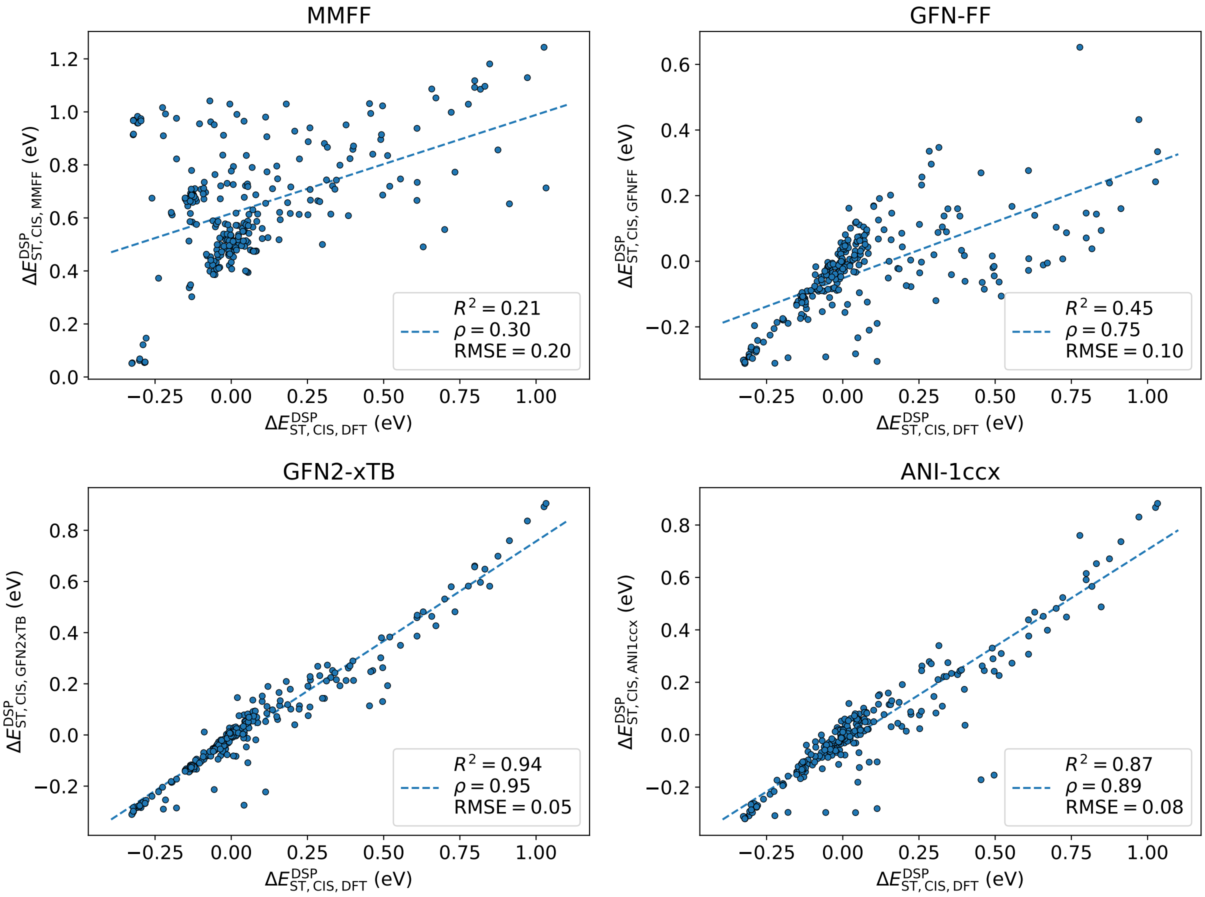

Figure S13: Singlet-triplet energy gaps with CIS + DSP for azaphenalenes at different geometries compared to DFT.

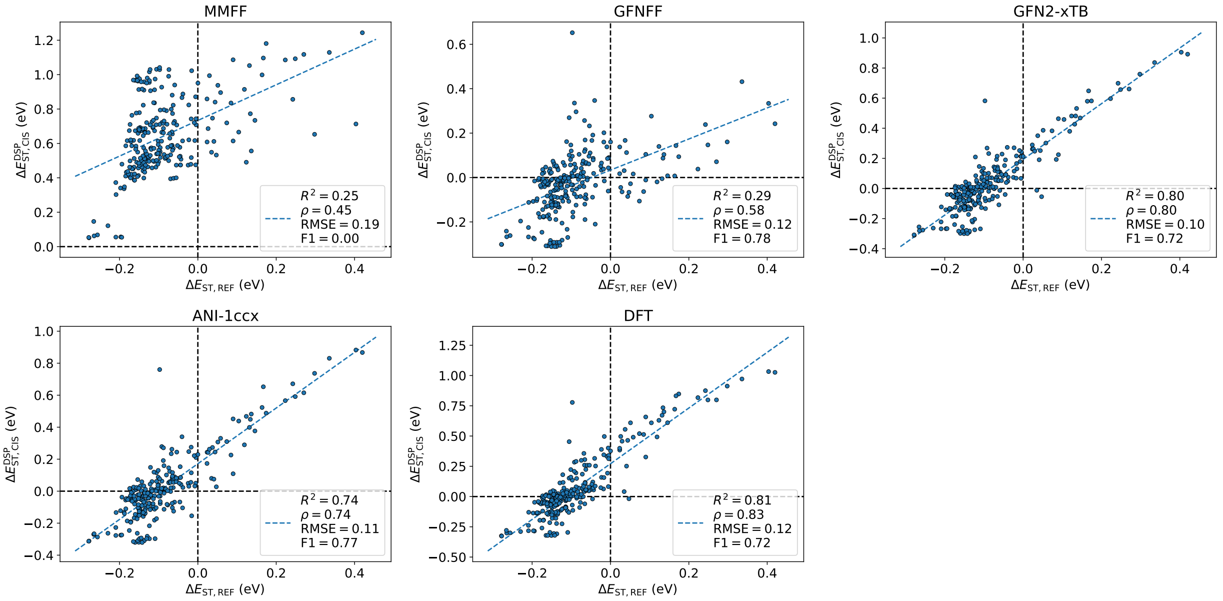

Figure S14: Singlet-triplet energy gaps with CIS + DSP against the reference level for azaphenalenes at different geometries.

References

(S1) Tharwat, A. Classification Assessment Methods. *Appl. Comput. Inform.* **2021**, *17* (1), 168–192. https://doi.org/10.1016/j.aci.2018.08.003.
